# Spatial referencing of chlorophyll fluorescence images for quantitative assessment of infection propagation in leaves demonstrated on the ice plant: *Botrytis cinerea* pathosystem

**DOI:** 10.1186/s13007-019-0401-4

**Published:** 2019-02-20

**Authors:** Joanna Sekulska-Nalewajko, Andrzej Kornaś, Jarosław Gocławski, Zbigniew Miszalski, Elżbieta Kuźniak

**Affiliations:** 1 Institute of Applied Computer Science, Lodz University of Technology, Stefanowskiego 18/22, 90-924 Lodz, Poland; 2 Institute of Biology, Pedagogical University, Podchorążych 2, 30-084 Kraków, Poland; 3 Institute of Plant Physiology, Polish Academy of Sciences, Niezapominajek 21, 30-239 Kraków, Poland; 4 Department of Plant Physiology and Biochemistry, Faculty of Biology and Environmental Protection, University of Lodz, Banacha 12/16, 90-237 Lodz, Poland

**Keywords:** Chlorophyll fluorescence, Image registration, PAM fluorometer, Pathogen infection

## Abstract

**Background:**

Chlorophyll fluorescence analysis is one of the non-invasive techniques widely used to detect and quantify the stress-induced changes in the photosynthetic apparatus. Quantitative information is obtained as a series of images and the specific fluorescence parameters are evaluated inside the regions of interest outlined separately on each leaf image. As the performance of photosynthesis is highly heterogeneous over a leaf surface, the areas of interest selected for generating numeric data are crucial for a reliable analysis. The differences in intact leaf physio-morphological characters and in the structural effects of stress between leaves increase the risk of artefacts.

**Results:**

The authors propose a new enhanced method for precise assessment of stress-induced spatiotemporal changes in chlorophyll *a* fluorescence exemplified in the leaves of common ice plants infected with a fungal pathogen. The chl *a* fluorescence leaf image series obtained with Imaging-PAM fluorometer are aligned both by affine and nonlinear spline transforms based on the set of control points defined interactively. The successive readings were taken on the same leaf and this image sequence registration allows to capture quantitative changes of fluorescence parameters in time and along selected directions on the leaf surface. The time series fluorescence images of attached leaf, aligned according to the proposed method, provide a specific disease signature for an individual leaf. The results for C_3_ and Crassulacean Acid Metabolism (CAM) plants have been compared with respect to the type of photosynthetic metabolism and the image alignment accuracy has also been discussed.

**Conclusions:**

The image alignment applied to the series of fluorescence images allows to evaluate the dynamics of biotic stress propagation in individual plant leaves with better accuracy than previous methods. An important use of this method is the ability to map the fluorescence signal horizontally in one leaf during disease development and to accurately compare the results between leaves which differ in morphology or in the structural effects of stress. This approach in analysing chlorophyll fluorescence changes can be used to receive spatial and temporal information over a sample area in leaves infected by different pathogenic fungi and bacteria.

**Electronic supplementary material:**

The online version of this article (10.1186/s13007-019-0401-4) contains supplementary material, which is available to authorized users.

## Background

In the photosynthesizing tissues/organisms, light energy absorbed by the chlorophyll molecules is primarily used to drive the energy-requiring reactions of photosynthesis (photochemistry). However, when absorbed in excess, it can be dissipated as heat or re-emitted as chlorophyll fluorescence. Most fluorescence comes from chlorophyll *a* (chl *a*) molecules associated with photosystem II (PSII). Although chlorophyll fluorescence represents only 1–2% of total light energy absorbed [1], in general its intensity is inversely proportional to the quantum of energy used for photosynthesis and to dissipative heat emission [2]. Therefore, using chlorophyll fluorescence many aspects of photosynthesis can be studied [1].

The plant photosynthetic apparatus is a sensitive sensor of most environmental stressors threating plant growth and crop production. Stress sensing is reflected in the imbalance of the photochemistry of PSII. Information on the state of PSII photochemistry can be obtained by chl *a* fluorescence analysis. Therefore, it has become a versatile, non-destructive and reliable method for the detection and quantification of abiotic and biotic stress-induced changes in the photosynthetic apparatus [1]. Chlorophyll *a* fluorescence analysis can be used in plant stress studies to detect the physiological response before any morphological stress signs become visible. This approach is widely used for small-scale as well as high throughput phenotyping studies [3].

Different types of fluorescence measurements are used in plant biology [4], with pulse amplitude modulation (PAM) fluorometry, being one of the most popular tools applied to monitor plant responses to environmental stresses. In PAM fluorometry, a high intensity light is switched on and off (pulse) at high frequency and the detector measures fluorescence emission only. This method allows to evaluate changes in fluorescence parameters in all photosynthetic organisms providing useful information about light energy transfer, photochemistry and non-radiative dissipation of the absorbed energy [5]. Techniques of automated chlorophyll fluorescence monitoring have been developed leading to an increase in the use of fluorimeters [3].

Fluorescence measurement systems provide the information in the form of images on quantum yield of photosynthetic energy conversion by specifying individual parameters, as for instance the maximal PSII quantum yield (F_v_/F_m_), quantum yield of photochemical energy conversion in PS II, Y(II), and the quantum yields of regulated and non-regulated energy dissipation in PSII, Y(NPQ) and Y(NO), respectively. Chlorophyll fluorescence provides images map on photosynthetic organs the variations of single fluorescence parameters in false-colour mode. The fluorescence parameters are usually quantified as the average values from identified stress regions, as for other computer-assisted image analyses [6, 7], obtained from manually-defined areas of interest. The performance of photosynthesis is highly heterogeneous over a single leaf surface and between leaves of a plant and the locally-specific changes driven by a stressor can be masked when the entire leaf surface is processed quantitatively. Therefore, areas of interest selected for generating numeric values for chlorophyll fluorescence are of key importance for a reliable analysis, especially when they cover veins and interveinal mesophyll cells, sites of low and high photosynthetic capacities, respectively. However, this problem receives insufficient discussion and the method for quantification of the heterogeneity of the fluorescence parameters is still lacking.

In this study, PAM fluorescence images have been used to investigate the spatiotemporal effects of fungal pathogen infection of the common ice plant leaves. The common ice plant, which undergoes a stress-induced transition from C_3_ mode of photosynthesis into Crassulacean Acid Metabolism (CAM), has become a model plant in the studies of the role of photosynthesis in stress tolerance. Both photosynthetic types differ in the activity of CO_2_-fixing enzymatic machinery; hence the activities of photosystems. The leaves of C_3_ and CAM-performing plants also differ with respect to the leaf morphology, physiology, pigment contents, leaf internal CO_2_ concentration and water availability, which influence the chlorophyll fluorescence [8]. Therefore, the interpretation of fluorescence data sets provided by the commonly employed procedures is sensitive to artefacts in the measured data.

To overcome the risk of artefacts due to the differences in intact leaf physio-morphological characters or in the structural effects of biotic damage between C_3_ and CAM plants, and due to the heterogeneity in the distribution of photosynthesis across the leaf area, we propose a method for the measurement of fluorescence parameters along the selected direction in a leaf blade image and in time scale. A new approach consists in analysing fluorescence changes in any defined leaf point or transect on archived images aligned for comparing the specific infection-induced spatiotemporal changes in chlorophyll fluorescence in more detail. This approach could be particularly important to maximize the information obtained from an experiment when two leaves differing in morphology or disease symptoms are paired to compare the effects of biotic stress or when a long-term fluorescence analysis of an individual leaf attached to a plant is performed. The image alignment technique, also known as the registration process, represents a group of numerical methods for transforming different images into one common coordinate system. The methods are based on the comparison of intensity, gradient in the whole images or on the matching regional features around specific control points selected according to various ideas [9, 10]. However, these popular, automatic methods fail when applied to the alignment of fluorescence leaf images (Additional file 1). Therefore, in this article a semi-automatic method has been proposed using matched pairs of control points defined interactively by specialists, as a new variation of the commonly used procedures for measuring chlorophyll fluorescence.

## Methods

### Plants and the pathogen

The common ice plant (*Mesembryanthemum crystallinum* L.) was grown in a greenhouse as described by Kuźniak et al. [11]. After the appearance of the 3rd leaf pair, one set of plants was irrigated with 0.4 M NaCl to induce Crassulacean Acid Metabolism (CAM plants) while another was further irrigated with tap water (C_3_ plants). After 12 days, the induction of CAM in the NaCl- treated plants was confirmed by measuring the diurnal ∆ malate in the leaf cell sap. Thereafter leaves of the 2nd leaf pairs of C_3_ and CAM plants were inoculated with *Botrytis cinerea* according to Kuźniak et al. [11].

### Chlorophyll *a* fluorescence imaging

The Mini version of an Imaging-PAM Chlorophyll Fluorometer M-Series (Walz, Effeltrich, Germany) equipped with a leaf holder was utilized to record fluorescence imaging (Imaging-PAM M-series Chlorophyll Fluorometer) [12]. The fluorometer is a leaf clip model for field applications. The leaf holder ensures that the leaf is held horizontal to the light source to avoid heterogenous illumination over different areas of the leaf sample. The sampling positions were chosen to be equally spaced along the midrib, however they slightly differed for any leaf due to its size and morphology, and the technique of placing leaves in the holder. To identify the effects of biotic stress only, and to avoid the introduction of artefacts in chlorophyll fluorescence measurement procedure, no reference marks allowing leaf image referencing were applied to the leaves. Chlorophyll fluorescence from common ice plant leaves was obtained by defining area of interests (AOI tool) using the Imaging Win 2.41a software.

With the Imaging-PAM, the current fluorescence yield (F_t_) was continuously monitored. Plants were dark adapted for 20 min. On application of a saturation pulse, the dark-level fluorescence yield (F_t_ = F_0_) and the maximum fluorescence yield (F_m_) were determined. The maximal PSII quantum yield, F_v_/F_m_, and the quantum yields of regulated and nonregulated energy dissipation in PSII, Y(NPQ) and Y(NO) were imaged. F_v_/F_m_ was calculated according to the equation: F_v_/F_m_ = (F_m_ − F_0_) F_m_. Y(NPQ) was calculated according to Kramer et al. [13] by the formula: 1 ‒ Y(II) ‒ 1/(NPQ + 1 + qL (F_m_/F_0_ − 1)). Y(NO) was calculated according to Kramer et al. [13] by the equation: Y(NO) = 1/[NPQ + 1 + qL (F_m_/F_0_ − 1)]. The process of imaging provides pseudo-colour (indexed color mode) images of biological material with a resolution 640 × 480 pixels corresponding to the view field of the physical dimensions 32 × 24 mm.

**Fig. 1:**
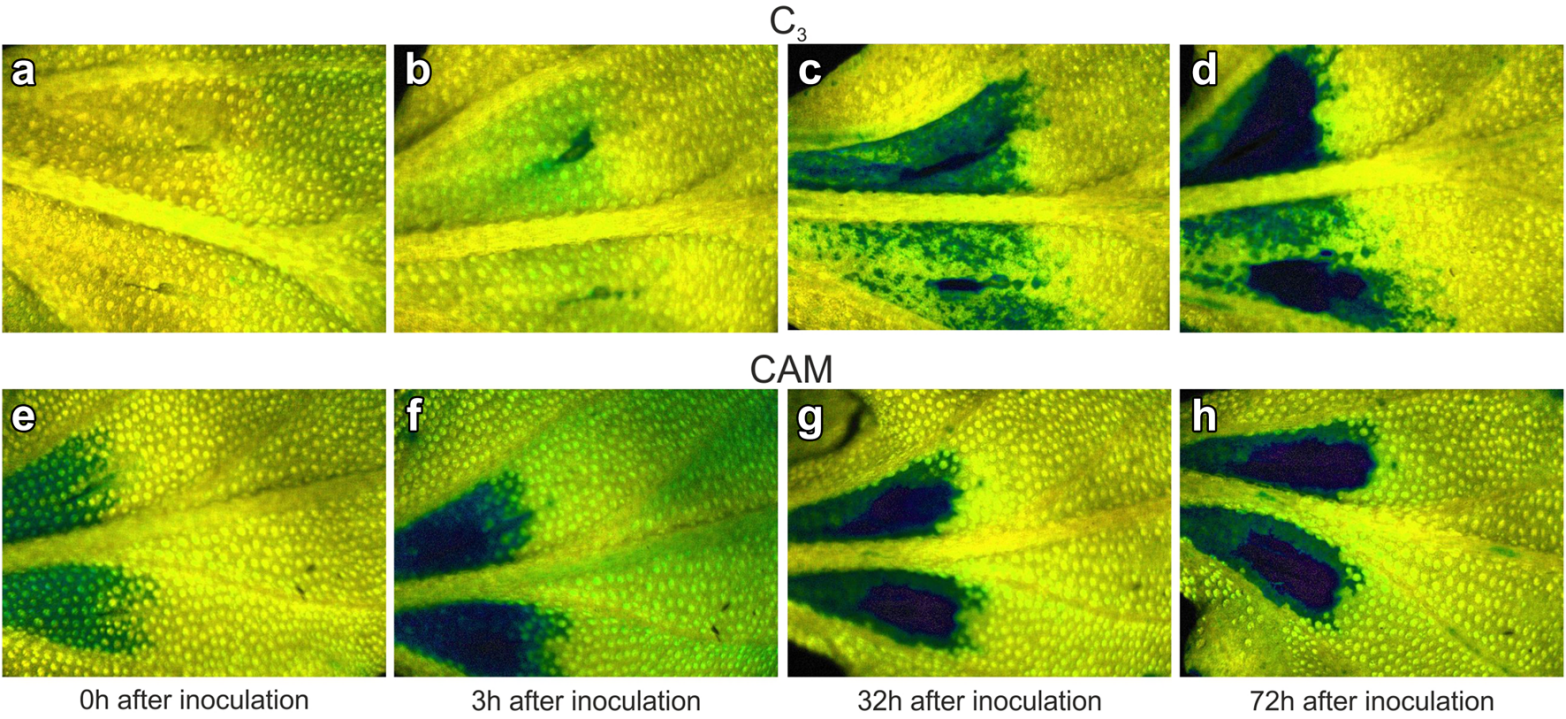
Example images of quantum yield of nonregulated energy dissipation in common ice plant leaves. Applies to PSII Y(NO) of C_3_ (**a**–**d**) and CAM (**e**–**h**) fluorescence parameters. The leaf fragment contains the site of pathogen inoculation and symptoms of stress propagation

**Fig. 2:**
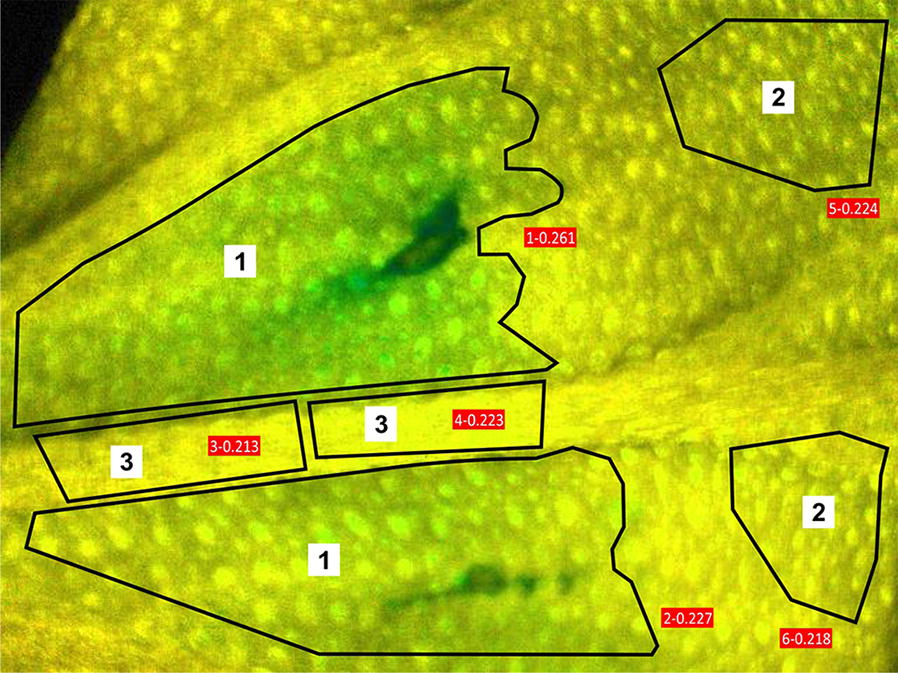
Example image of C_3_ common ice plant leaf with selected regions of interest. Manually made selections in firmware editor cover the infected (1), symptomless (2) as well as the midrib areas (3)

The infected leaves of the C_3_ and CAM plants were taken for chl *a* fluorescence analysis at the time point of inoculation and 3, 6, 9, 24, 32, 48, 54 and 72 h after inoculation. Chl *a* fluorescence was measured for attached leaves of the 2nd leaf pair from three C_3_ and CAM plants coming from two independent repetitions of plant cultivation. Each leaf taken for analysis was separately screened at all time points (Fig. 1). Representative series of nine images (one for each time point) of Y(NO), Y(NPQ) and F_v_/F_m_ for C_3_ and CAM ice plants have been processed to measure changes of these parameters in a selected area of leaf blade screened over time. For comparison, Y(NO), Y(NPQ) and F_v_/F_m_ averaged data from the entire leaf regions depicted in Fig. 2 were obtained. The results of image alignment and the measurement of the spatiotemporal pattering of biotic stress propagation in leaves with proposed method was exemplified on Y(NO) images.

### Image alignment

The mechanism of PAM image data collection in time sequence results in moving of the leaf fragment in the field of view between individual time points (Fig. 1). Due to the change of leaf position such characteristics as the midrib, and the inoculation sites have both different location and orientation. Moreover, the effect of rescaling can be observed. To proper assess stress propagation the view fields should be mutually synchronized assuming one of them as a reference (fixed) image and the rest of images to be aligned with the fixed one. This approach is known in medical imaging as image registration [14, 15]. The basic registration task for the fluorescence images is to find the ‘similarity’ transformation consisting of appropriate rotation, translation and scaling. The leaf surface bending and folding of leaves from stress occur only in local regions of single images and are of minor importance for the global image alignment. They can be compensated at the stage of registration postprocessing by nonlinear transforms like e.g. thin-plate, surface splines and demon mappings [9].

The fluorescence images are taken as a series of shots in predefined time intervals of approximately the same leaf region. The characteristic elements of considered stack of images represent mainly leaf veins. However they are poorly distinguishable from the image content due to limited contrast as well colour fashion of PAM imaging. The areas of stress symptoms visually dominating the contents may change between images in a single series. In such situation automatic registration would fail.

The most popular, automatic state of art methods are based on the comparison of image intensity with some correlation metrics (intensity-based methods) [9, 16] or rely on searching in the fixed and moving images for the correspondence between selected image features such as points, lines, and contours (feature-based methods) [10, 16–21]. None of these approaches allows for proper registration of PAM fluorescence images of common ice-plant, what has been verified and shown in the examples included in supplementary materials (Additional file 1). The results presented there confirm that the reason for unsuccessful automatic registrations is the strong obscuration of image regions with preserved features by dynamic changes in the image content caused by infection of the leaf tissue. Tests of the popular methods were carried out both by the *Registration Estimator* in Matlab and in the *Fiji* environment [22–26].

**Fig. 3:**
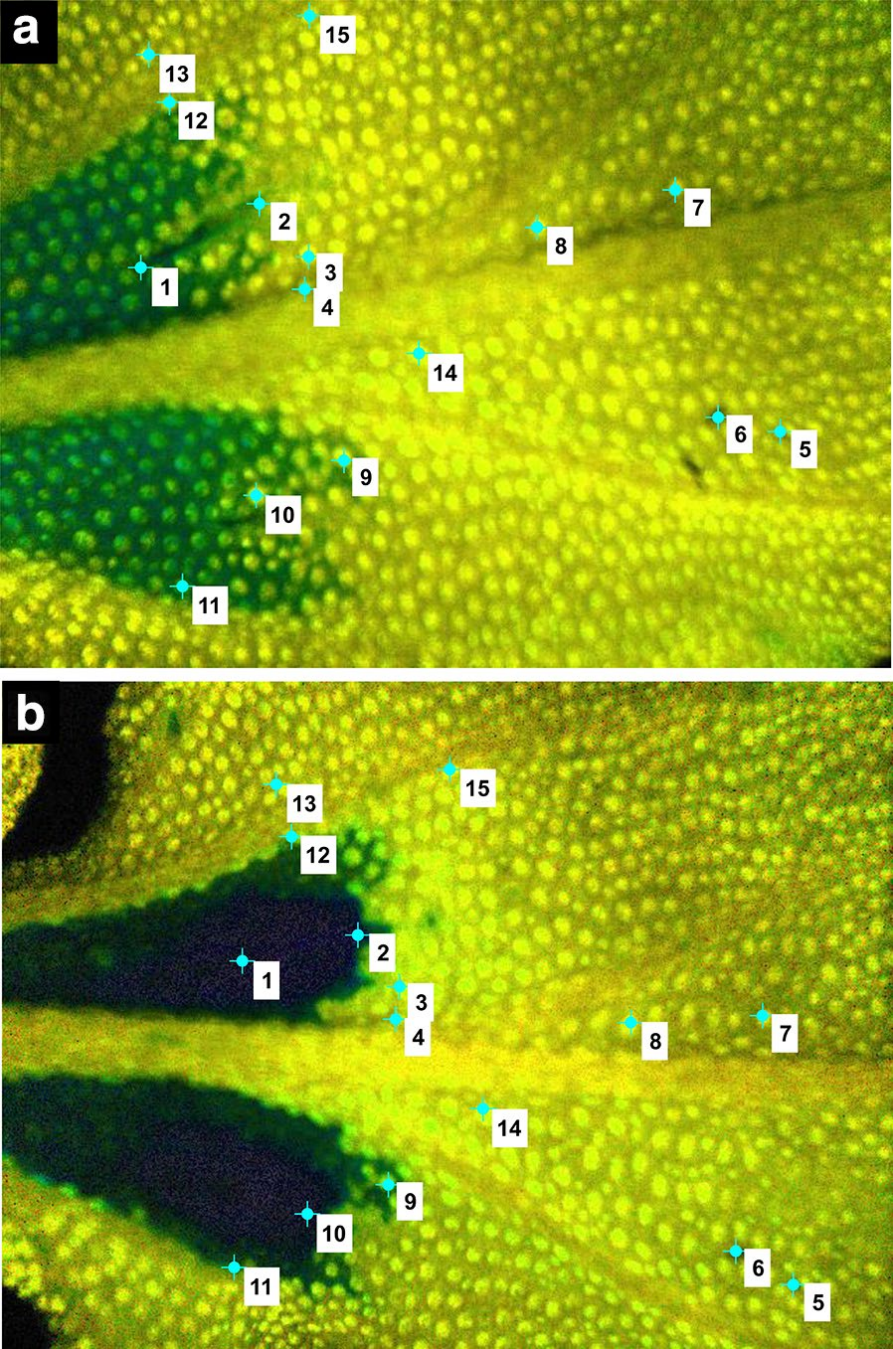
Control points selected by the expert. Example Y(NO) images of a leaf fragment of a CAM common ice plant: **a** fixed (reference) image, **b** moving image to be aligned with the fixed one

The algorithm of PAM image registration was designed in *Matlab* environment based on the set of control points selected manually by an expert. To set the corresponding control points in every image the function *cpselect* of *Control Point Selection Tool* from *Image Processing Toolbox* was applied. Images were edited in pairs including a fixed image and one moving image. The first image acquired just after pathogen inoculation was assumed as a fixed (reference) image. Through interactive point-mapping, the user can point not only the visible outlines of nerves, but also other characteristic elements such as the injection point, sites along the pathogen propagation as well as the most apparent epidermal bladder cells (Fig. 3).

**Fig. 4:**
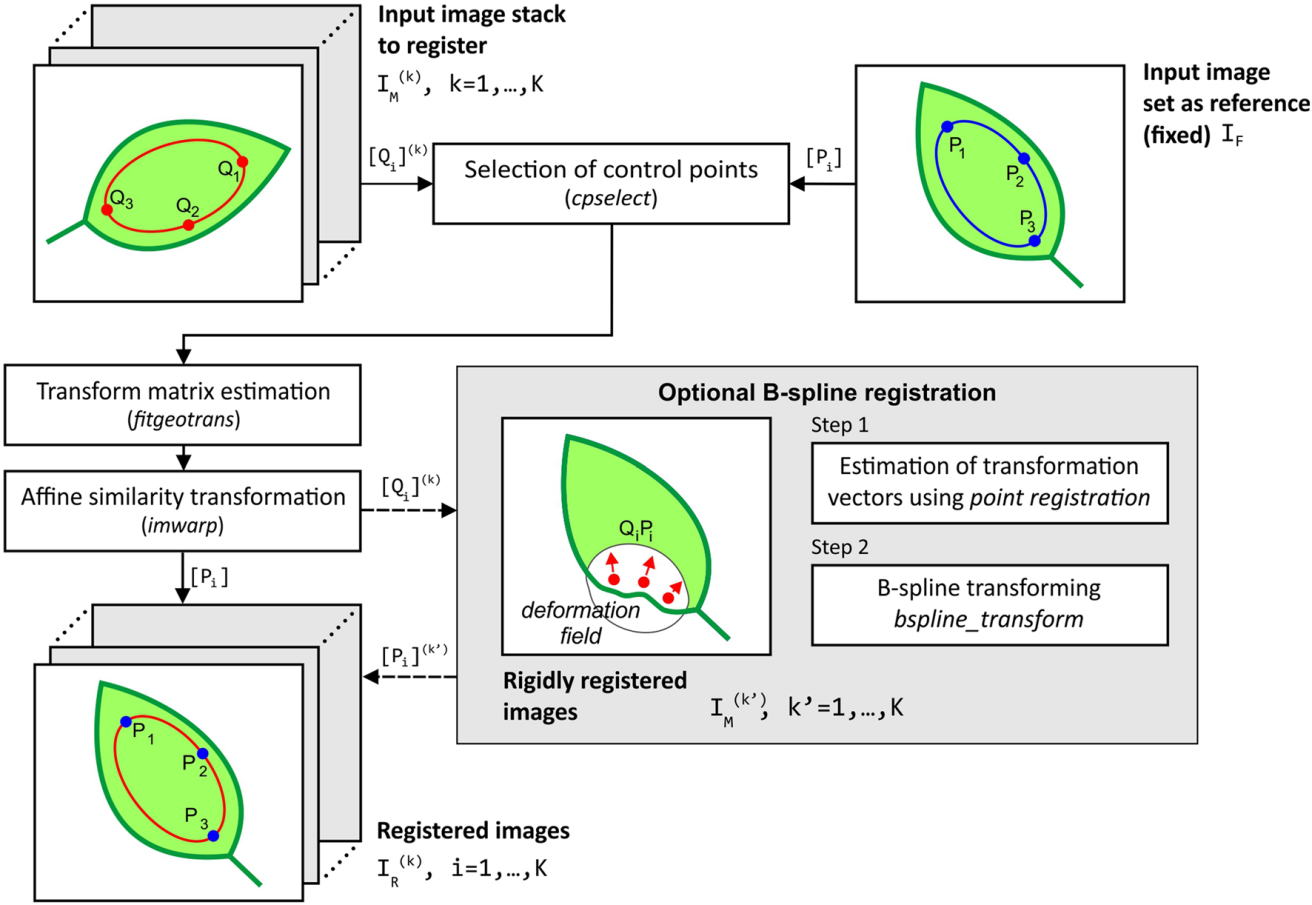
The block diagram of affine and optional B-spline registration applied to fluorescence PAM images. $\left[ {P_{i} } \right]$—the vector of control points in the fixed image, $\left[ {Q_{i} } \right]^{\left( k \right)}$—the vector of control points in *k*-th moving image, $\left[ {P_{i} } \right]^{{\left( {k^{\prime } } \right)}}$—the vector of control points in *k*-th moving image after B-spline registration

Several pairs of control point locations, distributed as widely as possible over the leaf image surface, are sufficient to properly align the images of the same leaf roughly considered as a rigid body. Wider distribution of control points improves the sensitivity of image matching but is limited by the image view field cropped after the alignment transform and by the possibility of precise location of the selected point on the leaf blade. With such image alignment the affine transform was performed using the function *fitgeotrans* included in *Image Processing Toolbox.* This transform has been limited to the ‘similarity’ version (consisting only of translation, rotation and similarity) because the scene in the PAM fluorometer appeared as not tilted. Moving images were matched to the fixed image using the function *imwarp*. Graphic illustration of the registration algorithm is included in Fig. 4.

**Fig. 5:**
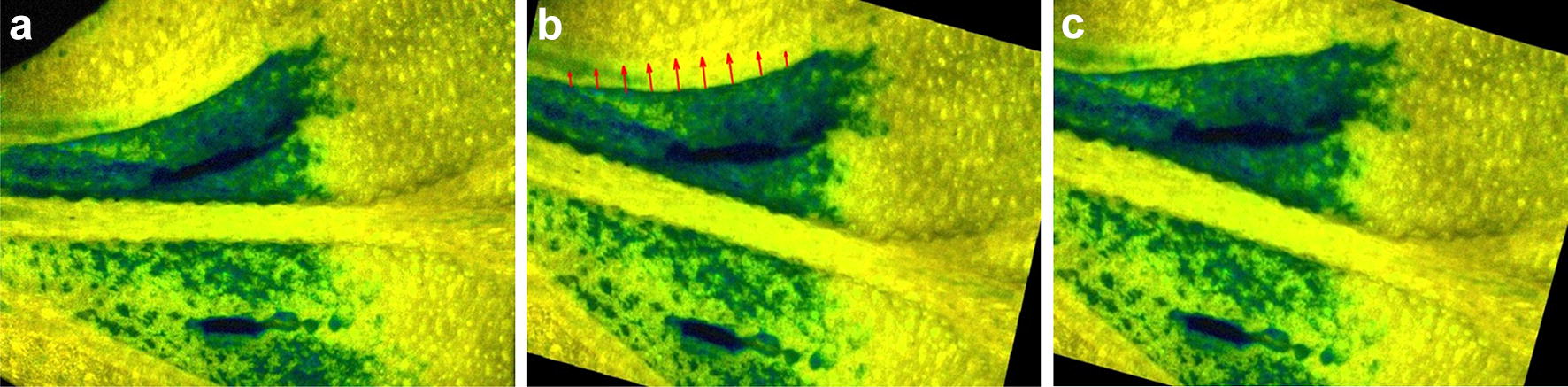
Example of affine and B-spline registrations for C_3_ common ice plant leaf image. **a** The Y(NO) image after PAM acquiring, **b** the image after rigid affine transformation with the arrows representing interactively set displacement vectors used in the second stage of registration and **c** final form after the control point-based B-spline registration

Leaves at various stages of infection may have a surface locally undulated or wrinkled like in the region marked in analysed PAM-fluorometer images (Fig. 5a, b), which shape can potentially influence the proper analysis of the pathogen propagation phenomenon. It means that the affine registration method based on geometric transformation may not be sufficient to match fluorescence PAM images in some cases of leaves with apparently visible nonlinear deformation. Therefore, the authors propose a two-stage registration method where affine rigid registration is followed by B-spline registration reducing nonlinear deformations.

The selection of non-rigid registration type exploits the fact that common ice plant leaves represent a little flexible material and small bending forces keep smooth changes in a leaf surface profile. The specificity of this registration modification is that the vectors of image deformation field have to be imposed interactively. For this purpose, a dedicated vector field editor was attached to the algorithm. Only few displacement vectors in the moving image must be specified to register local and smooth deformations of a leaf surface as in Fig. 5b.

The B-spline Rueckert algorithm [27, 28] was selected for the second stage of the registration. Its implementation is available in *Matlab Central File Exchange* as the Dirk-Jan Kroon *Spline Registration Toolbox* [29].

The B-spline registration procedure consists of two basic steps:

- Initialization of a grid G of image points evenly distributed across the image surface with all deformation field vectors set to zero, and then computing a dense vector field *T* of deformations in the grid *G* by cubic B-spline interpolation of manually set control point displacement vectors $\left[ {Q_{i} P_{i} } \right]^{\left( k \right)}$. Both the grid and the associated transform field *T* are then iteratively refined in 4 steps to reduce grid node spacing. The transform *T* is prepared by the function *point_registration* from *Spline Registration Toolbox*.
- B-spline transforming of all pixel positions and bi-cubic interpolations of colour components in the moving (affine registered) image $I_{M}$ according to the spline smoothed deformation field.

### Image registration accuracy

**Fig. 6:**
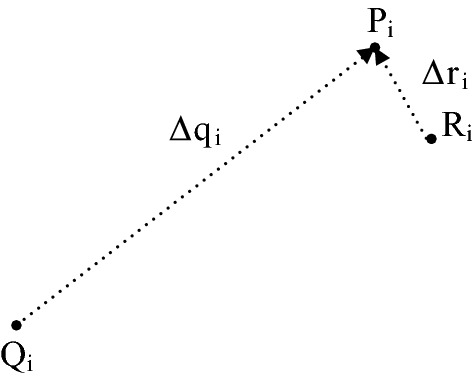
Illustration of the error assessment in affine registration of PAM images. $P_{i} ,\;i = 1, \ldots ,N$—control point in the fixed image $I_{F}$, $Q_{i}$—mapping of the control point $P_{i}$ in the moving image $I_{M}$, $R_{i}$—mapping of the control point $P_{\text{i}}$ after registration, $R_{i} P_{i} =\Delta r_{i}$—displacement error of the control point $P_{\text{i}}$ after registration

The accuracy of the proposed image alignment was performed by the root mean square (RMS) on deviations of *N *= 15 control points shown in Fig. 3. The displacement of each fixed image control point $P_{i}$ evaluated in a moving image for the case with and without the alignment were illustrated in Fig. 6 as the vectors $R_{i} P_{i} =\Delta r_{i}$ and $Q_{i} P_{i} =\Delta q_{i}$ respectively. The RMS displacement errors of all control points in a single image for the two cases are expressed in Eq. (1) as $\Delta r_{\text{rms}}$ and $\Delta q_{\text{rms}}$.

1 $$\Delta r_{\text{rms}} = \sqrt {\frac{1}{N}\mathop \sum \limits_{i = 1}^{N}\Delta r_{i}^{2} } ,\quad\Delta q_{\text{rms}} = \sqrt {\frac{1}{N}\mathop \sum \limits_{i = 1}^{N}\Delta q_{i}^{2} } .$$

The percentage of residual displacement $\delta_{rms}$ after affine type registration, can be evaluated per one image according to Eq. (2).

2 $$\delta_{\text{rms}} = \frac{{\Delta r_{\text{rms}} }}{{q_{\text{rms}} }}.$$

The evaluated affine registration errors are listed in Table 1. The original displacements $\Delta q_{\text{rms}}$ varying from 3.01 to 7.09 mm are reduced after the ‘similarity’ registration to the range from 0.45 to 1.71 mm of $\Delta r_{\text{rms}}$ for the tested C_3_ plant. The same parameters for CAM plant are $1.90 \div 5.69\;{\text{mm}}$ for $\Delta q_{\text{rms}}$ and $0.41 \div 1.53\;{\text{mm}}$ for $\Delta r_{\text{rms}}$. When $\delta_{rms}$ is averaged both for C3 and CAM image series, it equals about 21% and 23% respectively. The values of fluorescence parameters Y(NO), F_v_/F_m_ and NPQ obtained from ice-plant leaf images without registration are retrieved from incompatible parts of the leaf and cannot be taken into account (see Additional file 2).

**Table 1:** Errors of the control point displacements for C_3_ and CAM fluorescence ice plant leaf images

Hours post inoculation	C_3_			CAM		
	$\Delta q_{rms}$ [mm]	$\Delta r_{rms}$ [mm]	$\delta_{rms}$ [%]	$\Delta q_{rms}$ [mm]	$\Delta r_{rms}$ [mm]	$\delta_{rms}$ [%]
3	3.69	0.81	21.95	3.60	0.80	22.22
6	3.01	0.45	14.95	3.09	1.53	49.51
9	7.09	0.48	6.77	1.90	0.77	40.53
24	2.69	0.83	30.86	4.52	0.53	11.73
32	3.55	1.23	34.65	4.55	0.98	21.54
48	3.81	0.58	15.22	4.86	0.56	11.52
54	5.99	1.71	28.55	5.45	0.41	7.52
72	6.76	1.10	16.98	5.69	1.04	18.28

The images are acquired in time from 3 h to 72 h after inoculation, the errors related to the reference image taken just after inoculation. $\Delta q_{rms}$—RMS displacement of control points in an image without alignment, $\Delta r_{rms}$—RMS displacement of the control points after affine type registration, $\delta_{rms}$—percentage displacement error after affine registration

**Fig. 7:**
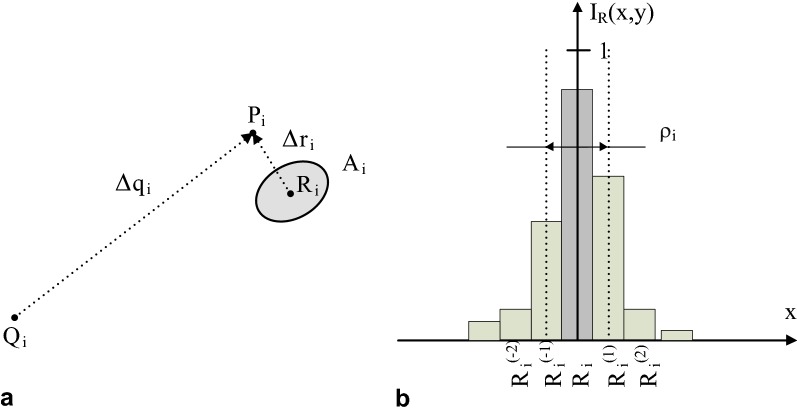
Explanation of the error in B-spline image registration of PAM images. **a** The mismatch of control point location mapping during registration, **b** image intensity distribution of spline registered control point, $P_{i} ,\;i = 1, \ldots ,N$—control point in the fixed image $I_{F}$, $Q_{i}$—the equivalent of control point $P_{i}$ in the moving image $I_{M}$, $R_{i}$—mapping of the control point $P_{i}$ after registration, $R_{i} P_{i} =\Delta r_{i}$—displacement error measure of mapping the control point $P_{i}$, $A_{i}$—blurred region around $R_{i}$ corresponding to the control point $P_{i}$, $\rho_{i}$—the standard deviation radius of $R_{i}$ blur

For additional B-spline registration mapping the transform error is defined by two components. The first of them is the post registration displacement $\Delta r_{i} = R_{i} P_{i}$ shown in Fig. 7a, with $R_{i}$ evaluated in Eq. (3) as the centroid $\bar{p}$ of the region $A_{i}$.

3 $$\bar{p} = \frac{1}{{\left| {A_{i} } \right|}}\mathop \sum \limits_{{p \in A_{i} }} I_{R} \left( p \right),\quad i = 1, \ldots ,N,$$

where $I_{R} \left( p \right) \in \left[ {0,1} \right]$ denotes the intensity of registered control point image at the pixel *p*, $\left| {A_{i} } \right|$—the blurring region area. The error second component is defined by the standard deviation radius $\rho_{i}$ of intensity spread around each control point $R_{i}$ as described in Eq. (4).

4 $$\rho_{i} = \sqrt {\frac{1}{{S_{i} }}\mathop \sum \limits_{{p \in A_{i} }} I_{R} \left( p \right)p - \bar{p}^{2} } ,\quad S_{i} = \mathop \sum \limits_{{p \in A_{i} }} I_{R} \left( p \right),\quad i = 1, \ldots ,N,$$

where $\left\| \cdot \right\|$ denotes the Euclidean norm of the vector between points *p* and $\bar{p}$ in the image plane. Blurring of the aligned control point $R_{i}$ appears due to the fact that the nonlinear registration uses bicubic interpolation in the finite resolution grid *G*. This effect was measured experimentally by performing a given B-spline transform in the image built from white control points on a black background. Table 2 includes the magnitudes $\Delta q_{i}$ of *N *= 9 example displacement field vectors shown in Fig. 5b. The magnitudes $\Delta r_{i}$ of the vector mapping errors after spline registration are measured between the desired fixed control points $P_{i}$ and the centroids of registered points $R_{i}$ evaluated in the region $A_{i}$. All tested $\Delta r_{i}$ values are below the pixel resolution equal to $50\;\upmu{\text{m}}$ and may be neglected—rounded to 0. This means precise positioning of control points by the spline transform. The standard deviation $\rho_{i}$ of blurring region shown in Table 2 after doubling can be a measure of control point blur. Then it varies approximately from $24\;{\text{to}}\;63\;\upmu{\text{m}}$ what is the equivalent of one-pixel blur. Thus this transform allows to locally restore the proper shape of Y(NO) changes in a fluorescence image.

**Table 2:** Errors of control point displacement for the example in Fig. 5

	CP number								
	1	2	3	4	5	6	7	8	9
$\Delta q_{i}$ [µm]	1410	1610	1520	1560	1170	1360	960	750	810
$\Delta r_{i}$ [µm]	4.74	6.99	11.35	2.20	5.64	1.84	3.35	11.21	6.39
$\rho_{i}$ [µm]	16.44	20.84	27.18	12.11	20.10	12.39	15.28	31.58	24.01

Applies to the C_3_ fluorescence common ice plant leaf image captured 32 h after infection. $\Delta q_{i}$—displacement of *i*-th control point (CP) without spline alignment, $\Delta r_{i}$—displacement of *i*-th control point maximum intensity after spline registration, $\rho_{i}$—the standard deviation radius of point blur

### The measurement of stress propagation

**Fig. 8:**
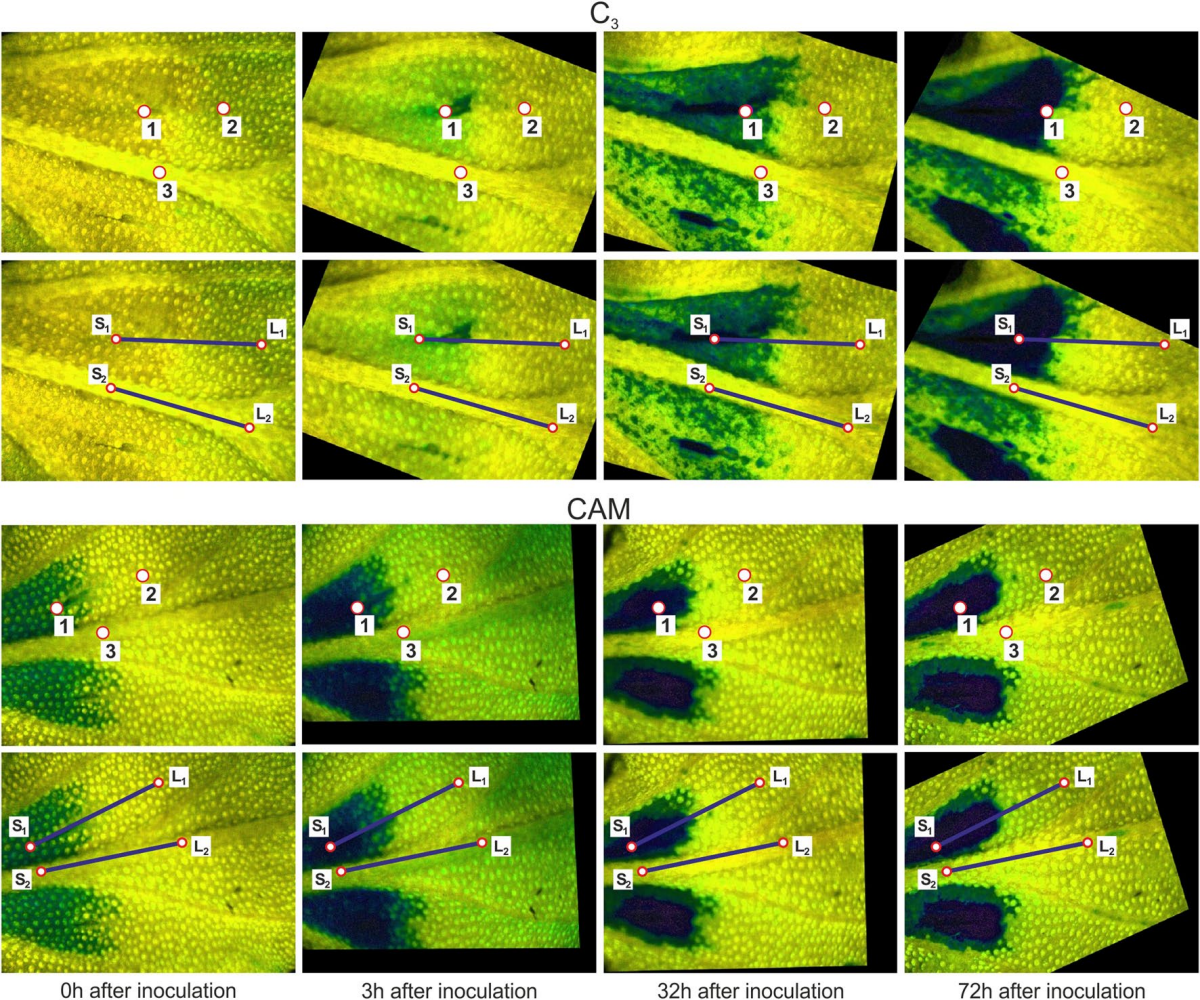
Measurement regions in Y(NO) images of common ice plant leaves after the computer alignment. The measurement points indicate: (1) mesophyll at the site of inoculation, (2) mesophyll without injury, (3) midrib near the inoculation site. The measurement line (*L*_*1*_) is oriented in the direction of stress propagation within mesophyll and the line (*L*_*2*_) is located along the midrib. Lines started at points (*S*_*1*_) and (*S*_*2*_), respectively

The data analysis applies a special editor tool for manipulating a stack of PAM images after their registration. The main editor function allows drawing two data acquisition line sections $L_{1} \;{\text{and}}\;L_{2}$ of equal length (Fig. 8), which can be observed and available on any image from the stack. In the considered experiment the first line $L_{1}$ starts at the site of inoculation $s_{1}$, where the stress factor is applied to the leaf tissue at time *t*, and should be approximately set in the direction of stress expansion. The second line $L_{2}$ is placed along the midrib where the observed stress influence in time was always limited and fluorescence parameters exhibit minimal changes. The lines should fit entirely in the context of the image.

Additional option of point-wise measurement is possible where three small different circular regions of the 10 pixels radius are placed interactively in the field of view (Fig. 8). They should belong to the leaf regions with different fluorescence parameter pattering over time. All registered pixels values are averaged inside these regions.

## Results and discussion

The photosynthetic performance of plants under biotic stress is commonly assessed from F_v_/F_m_ and NPQ [3, 30, 31], and not much information is available on the meaning of Y(NO). Changes in Y(NO) are complementary to those of PSII quantum yield of photochemical energy conversion, Y(II) and Y(NPQ). These three parameters provide information on the fate of absorbed light energy. Y(NO) reflects the fraction of light energy that is passively dissipated in form of heat and fluorescence, mainly due to closed PS II reaction centres. A high Y(NO) value indicates that both photochemical energy conversion and protective regulatory mechanisms (NPQ) are inefficient, and the plant is at risk from photodamage leading to the physical damage of PSII reaction centres [32].

### Infection-induced changes in chlorophyll a fluorescence in C_3_ and CAM plants

The quantification of chl *a* fluorescence changes from the entire leaf regions showed that pathogen infection differentially affected chlorophyll fluorescence in the infected leaf area close to the inoculation sites, in the undamaged leaf mesophyll and near the midrib (Table 3).

**Table 3:** Changes in quantum yield of nonregulated energy dissipation

Plants	C_3_						CAM					
Hours post inoculation	0	3	6	9	24	48	0	3	6	9	24	48
F_v_/F_m_												
Infected leaf area	0.70 ± *0.01*	0.64 ± *0.02*	0.50 ± *0.04*	0.52 ± *0.03*	0.50 ± *0.08*	0.58 ± *0.06*	0.62 ± *0.00*	0.41 ± *0.03*	0.50 ± *0.04*	0.56 ± *0.01*	0.50 ± *0.08*	0.58 ± *0.07*
Midrib	0.67 ± *0.01*	0.68 ± *0.01*	0.60 ± *0.00*	0.62 ± *0.00*	0.65 ± *0.01*	0.72 ± *0.00*	0.66 ± *0.01*	0.61 ± *0.02*	0.60 ± *0.00*	0.65 ± *0.00*	0.64 ± *0.01*	0.72 ± *0.00*
Non- infected leaf area	0.66 ± *0.02*	0.63 ± *0.01*	0.56 ± *0.04*	0.58 ± *0.02*	0.62 ± *0.02*	0.69 ± *0.01*	0.69 ± *0.00*	0.59 ± *0.00*	0.57 ± *0.04*	0.63 ± *0.01*	0.62 ± *0.02*	0.69 ± *0.02*
NPQ												
Infected leaf area	0.17 ± *0.01*	0.18 ± *0.00*	0.16 ± *0.01*	0.15 ± *0.01*	0.12 ± *0.02*	0.05 ± *0.01*	0.05 ± *0.00*	0.04 ± *0.00*	0.16 ± *0.01*	0.14 ± *0.01*	0.12 ± *0.02*	0.05 ± *0.02*
Midrib	0.20 ± *0.00*	0.17 ± *0.00*	0.19 ± *0.01*	0.20 ± *0.01*	0.18 ± *0.00*	0.07 ± *0.01*	0.16 ± *0.01*	0.20 ± *0.03*	0.19 ± *0.00*	0.18 ± *0.00*	0.18 ± *0.00*	0.07 ± *0.01*
Non- infected leaf area	0.17 ± *0.00*	0.26 ± *0.01*	0.26 ± *0.00*	0.32 ± *0.02*	0.29 ± *0.03*	0.16 ± *0.03*	0.15 ± *0.01*	0.18 ± *0.00*	0.24 ± *0.00*	0.23 ± *0.00*	0.29 ± *0.03*	0.17 ± *0.02*
Y(NO)												
Infected leaf area	0.37 ± *0.02*	0.35 ± *0.01*	0.44 ± *0.04*	0.46 ± *0.03*	0.52 ± *0.07*	0.45 ± *0.08*	0.64 ± *0.01*	0.75 ± *0.01*	0.44 ± *0.03*	0.46 ± *0.04*	0.52 ± *0.07*	0.45 ± *0.09*
Midrib	0.36 ± *0.02*	0.33 ± *0.02*	0.36 ± *0.01*	0.33 ± *0.01*	0.36 ± *0.02*	0.27 ± *0.01*	0.35 ± *0.00*	0.34 ± *0.01*	0.36 ± *0.01*	0.34 ± *0.02*	0.37 ± *0.02*	0.26 ± *0.01*
Non- infected leaf area	0.35 ± *0.02*	0.28 ± *0.01*	0.33 ± *0.02*	0.28 ± *0.00*	0.29 ± *0.02*	0.23 ± *0.01*	0.37 ± *0.02*	0.37 ± *0.00*	0.33 ± *0.02*	0.33 ± *0.00*	0.29 ± *0.02*	0.22 ± *0.00*

In PSII, Y(NO) measured on leaves of C_3_ and CAM common ice plants. Shown are average values (± SD) from the selected leaf areas

The value of F_v_/F_m_ was temporarily decreased, especially within the region covered by infection symptoms. At this location, the regulated protective mechanisms (NPQ) were activated only in CAM plants, shortly after inoculation (6–24 h). Interestingly, NPQ increased in the non-infected mesophyll of C_3_ and CAM leaves up to 24 h after inoculation, indicating that the biotic stress was signalled to the leaf regions distant from the inoculation site, where no visible injury was observed. In the leaf area covered with infection symptoms, Y(NO) increased and compensated for the low NPQ. In CAM plants, this effect was visible shortly after inoculation (3 h) whereas in C_3_ plants it was found after 24 h and 48 h (Table 3). In the regions distant from the inoculation sites, not covered by the infiltrated inoculum, and in the midrib, Y(NO) remained roughly constant. These results indicated that the necrosis of infected areas which appeared 2–3 days after inoculation [11], could be caused by the depression (C_3_, Table 3) or insufficient mobilization (CAM, Table 3) of the regulated photoprotective mechanisms (NPQ) around the sites of inoculation, leading to irreversible damage of PSII.

**Fig. 9:**
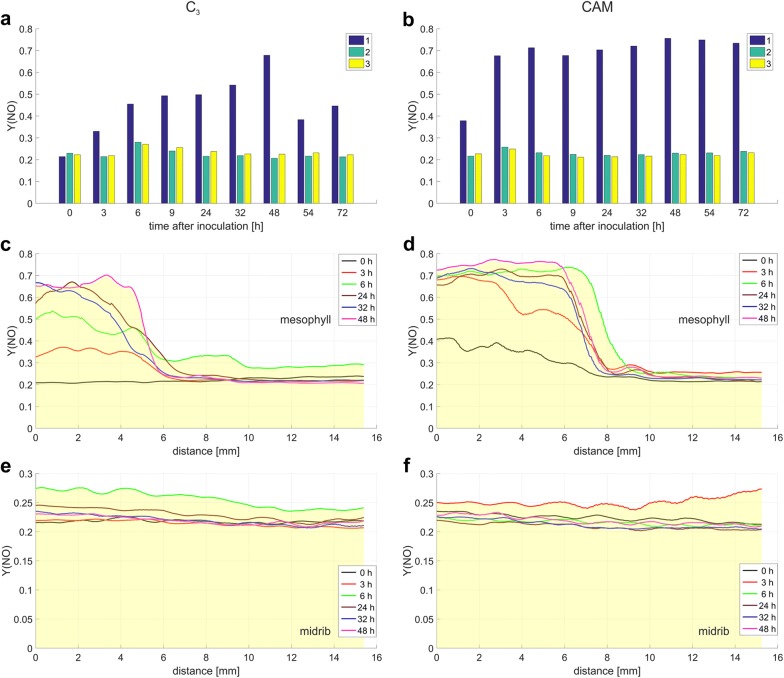
The Y(NO) value changes in C_3_ and CAM common ice plant leaves. Y(NO) values are computed at three locations (**a**, **b**) and along the line distance *L*_1_ and *L*_2_ (**c**–**f**) depicted in (**a**, **b**). The measurement points are: (1) mesophyll at the site of inoculation, (2) mesophyll without injury, and (3) midrib; **c**, **d** mesophyll at the site of inoculation; **e**, **f** midribs

**Fig. 10:**
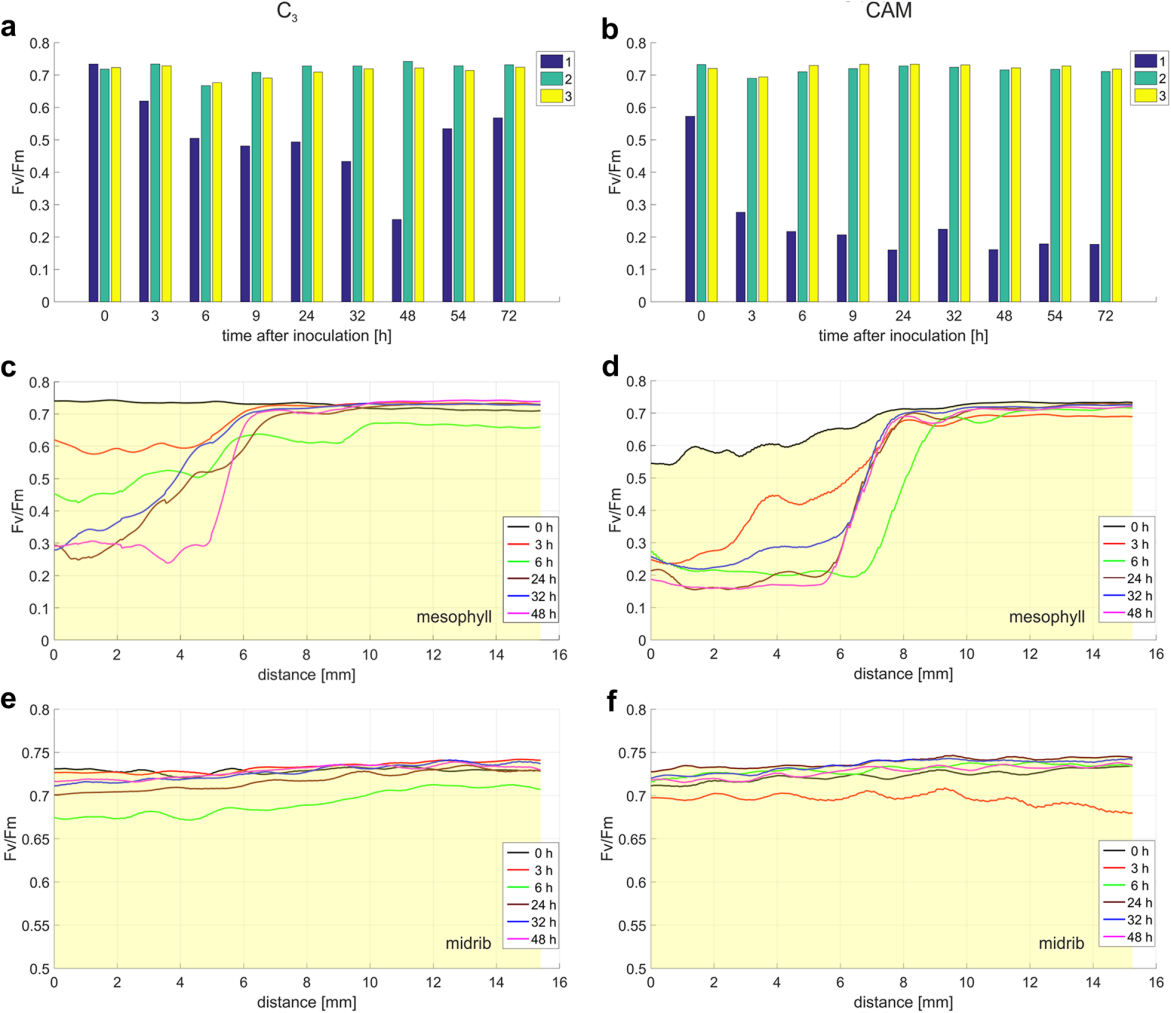
The fluorescence parameter F_v_/F_m_ value changes in C_3_ and CAM common ice plant leaves. F_v_/F_m_ is computed at three locations (**a**, **b**) and along the line distance *L*_1_ and *L*_2_ (**c**–**f**) depicted in Fig. 9a, b. The measurement points are: (1) mesophyll at the site of inoculation, (2) mesophyll without injury, and (3) midrib; **c**, **d** mesophyll at the site of inoculation; **e**, **f** midribs

**Fig. 11:**
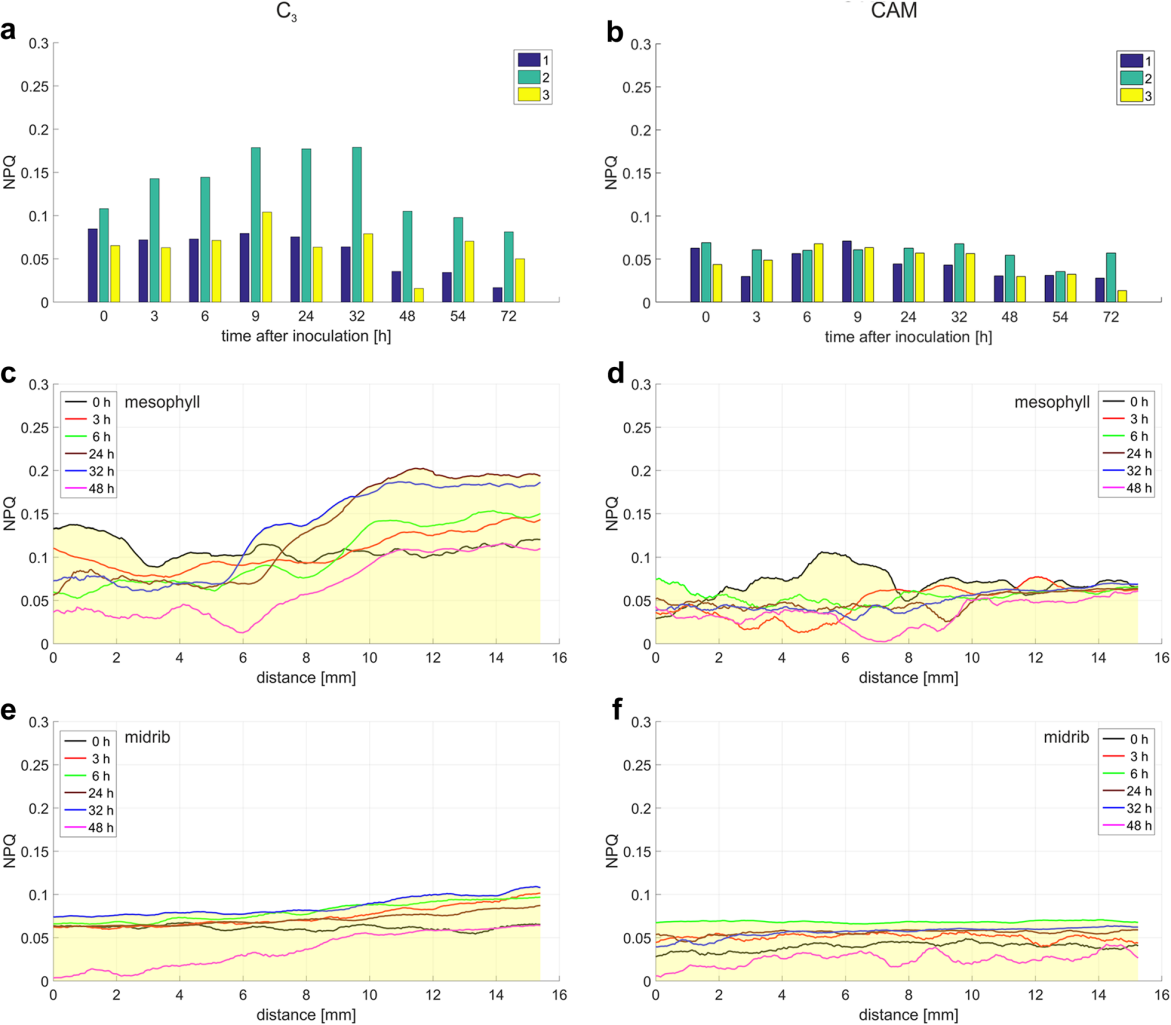
The fluorescence parameter NPQ value changes in C_3_ and CAM common ice plant leaves. NPQ is computed at three locations (**a**, **b**) and along the line distance *L*_1_ and *L*_2_ (**c**–**f**) depicted in Fig. 9a, b. The measurement points are: (1) mesophyll at the site of inoculation, (2) mesophyll without injury, and (3) midrib; **c**, **d** mesophyll at the site of inoculation; **e**, **f** midribs

The new approach to assess the impact of biotic stress by using chl *a* fluorescence measurement described herein, is exemplified by application to the quantification of Y(NO) changes. The time-dependent quantification of Y(NO) on individual spots (Fig. 8) selected on the fluorescence images of a given leaf aligned according to the proposed method, revealed different profiles of Y(NO) signal propagation than those obtained for the entire leaf regions. We found that in the midrib and mesophyll tissue distant from the inoculation sites, Y(NO) value was maintained at the level of about 0.2 (Fig. 9a, b), reported for healthy green leaves [31]. At the inoculation sites Y(NO) increased significantly, especially in CAM plants. These data on the Y(NO) response in the damaged leaf area, and the F_v_/F_m_ and NPQ data likewise, differed from the averaged ones with respect to the dynamics and in relation to the photosynthetic metabolism. Within the symptomless mesophyll regions and the midribs, the differences between the averaged F_v_/F_m_, NPQ and Y(NO) data and those collected from individual spots were subtler (Figs. 9, 10, 11). In comparison to the commonly employed procedure, the new approach provides more accurate estimation of the infection effects on the photosynthetic apparatus emphasizing the difference between C_3_ and CAM plants.

The distance that the increased Y(NO) extended away from the inoculation site over a 48-h period was significantly longer in the infected leaves of CAM plants than in C_3_ ones (Fig. 9). A maximum distance of the Y(NO) wave propagation was observed 6 h after inoculation. At this time point, it was longer (although less intense) in C_3_ than in CAM plants. These results, especially when accompanied with the analysis of the waves of F_v_/F_m_ and NPQ (Figs. 10, 11), can provide further information on the fluorescence signature induced by biotic stress in C_3_ versus CAM plants and on the dependence of the plant defence response on the type of photosynthetic metabolism. The physiological meaning of the Y(NO) wave observed here appears one of the interesting aspects of stress signalling which could be investigated.

The fluorescence parameters are usually quantified as the average of random measurements or of manually-defined large-sized areas within several leaf blades. Biological replication would be useful if similar patterns of stress response occur. Averaging the data for several leaves, however, would invalidate the full potential of the established method as it did not provide detailed data on the spatial heterogeneity of the stress effects. Therefore, it may underestimate the impact of a pathogen on photosynthesis in the infected plants. In this study, the area of infection-induced irreversible damage of the photosynthetic apparatus, reflected by an increase in Y(NO), was identified by using precisely aligned images. This approach also provided a detailed description of the unique fluorescence wave spreading from the inoculation sites. In plant ecophysiology and stress-physiology, this approach can be particularly advantageous when the damage effects are spatially heterogeneous and the objects to be compared show phenotypic differences.

CAM plants demonstrated increased leaf and mesophyll thickness as well as cell size and succulence, accompanied by reduced intercellular air space relative to C3 plants [33]. These anatomical traits did not change the robustness of the algorithm as the accuracy of the registration algorithm is independent on the image contents. It is associated with the correct distribution and the number of control point pairs introduced in compared images. When selecting control points their uniform distribution in the image should be provided. Increasing the number of control point pairs above the minimal 3 can be the factor improving the ‘similarity’ alignment accuracy, but this effect disappears above 10–15 points, which number was selected in the presented algorithm. The image content and biological scale of the image can influence on the human precision during the placement of control points, but the precision scale cannot be defined or measured by mathematical methods.

Image registration primarily uses automatic registration based on unchanging elements of image contents especially edges and characteristic dark or bright areas. This approach is known in medicine and is often applied for magnetic resonance imaging (MRI) and computed tomography (CT) 3-dimensional images. Medical registration primarily uses automatic registration based on unchanging elements of image contents especially edges and characteristic dark or bright areas [9]. In the leaf image, the best source of reference points can be the blade border [34]. Although the common ice plant images contain two-dimensional leaf fragments, there are no unambiguous anatomical structures which could be the basis for automatic registration. According to [35] for automatic registration of two-dimensional images reference points can be introduced directly to the leaf. However, in the case of chlorophyll fluorescence markers put on the leaf blade, especially with the use of chemical substances, this may cause side effects manifested by fluorescence disturbances.

Chlorophyll *a* fluorescence images have been shown to be useful to characterize plant response to environmental stresses [2, 3]. Although described for one pathosystem, this approach can be used to monitor all diseases that appear on leaves as spots of various sizes and types, e.g. bacterial spots caused by *Pseudomonas* and *Xanthomonas*, anthracnose and rust caused by fungi as well as non-infectious diseases induced by abiotic environmental factors such as air pollutants. Moreover, chlorophyll fluorescence imaging can be integrated with other non-invasive spectroscopy-based methods such as thermography, reflectance and near-infrared imaging which enables better analysis of the sample area [36].

## Conclusions

This paper presents a computer method based on the measurements of chl *a* fluorescence parameters, that allow to evaluate the dynamics of biotic stress propagation in plant leaves in the selected direction on the leaf blade. Image alignment and automation of measurements provide more accurate and objective analyses by ensuring that the results are always read at the same location within leaves.

The time series fluorescence images taken on an attached leaf and aligned according to the proposed method, provide a specific disease signature of an individual leaf. This approach, providing spatiotemporal information over a sample area, is well suited to compare the localized disease symptoms between leaves, especially when the spot lesions appear asynchronously, and to elucidate the relationship between the photosynthetic pathways of carbon assimilation of C_3_/CAM types and plant response to infection. This method can be applied to all fluorescence parameters obtained by PAM fluorometer when it is needed to extract as much detailed information as possible from limited data sets.

## Additional files

**Additional file 1**. Illustration of the results of tests for automatic registration of fluorescence images. **Figure** **1.** Input image pairs to register: fixed image (on the left) and moving image (on the right). **Figure** **2.** Intensity based monomodal ‘similarity’ registration with gradient descent optimizer. **Figure** **3.** ‘Similarity’ registration with 2D phase correlation algorithm. **Figure** **4.** ‘Similarity’ registration using the automatic detection of SURF (*Speeded*-*Up Robust Features*). **Figure** **5.** ‘Similarity’ registration based on the algorithm of automatic detection and matching MSER features (*Maximally Stable Extremal Regions*). **Figure** **6.** ‘Similarity’ registration with the Fiji (ImageJ) plugin *Linear Stack Alignment with SIFT*. **Figure** **7.** ‘Similarity’ registration with the Fiji (ImageJ) plugin *Register Virtual Stack Slices* aided by automatically extracted SIFT features (*Scale Invariant Feature Transform*). **Figure** **8.** Examples of ‘similarity’ registration with the Fiji (ImageJ) plugin *Descriptor*-*based registration* (2*d/3d*) using SPIM (*Selective Plane Illumination Microscope*) method.

**Additional file 2**. The values of fluorescence parameters Y(NO), F_v_/F_m_ and NPQ obtained from ice-plant leaf images without registration. **Figure** **1.** The fluorescence parameter Y(NO) value changes in C_3_ and CAM common ice plant leaves obtained from ice-plant leaf images without registration. **Figure** **2.** The fluorescence parameter F_v_/F_m_ value changes in C_3_ and CAM common ice plant leaves obtained from ice-plant leaf images without registration. **Figure** **3.** The fluorescence parameter NPQ value changes in C_3_ and CAM common ice plant leaves obtained from ice-plant leaf images without registration.

## Abbreviations

AOI: area of interest
CAM: Crassulacean Acid Metabolism
Chl *a*: chlorophyll *a*
CP: control points
CT: computed tomography
MRI: magnetic resonance imaging
NPQ: non-photochemical quenching
PAM: pulse amplitude modulation
PS II: Photosystem II
RMS: root mean square
SD: standard deviation
