# Supplementary material for: Spatial referencing of chlorophyll fluorescence images for quantitative assessment of infection propagation in leaves demonstrated on the ice plant: Botrytis cinerea pathosystem
Source: Plant Methods. 2019 Feb 20;15:18. doi: 10.1186/s13007-019-0401-4 (PMC6381734; doi:10.1186/s13007-019-0401-4)
Supplement: Supplementary file 1 — Additional file 1. Illustration of the results of tests for automatic registration of fluorescence images. Figure 1. Input image pairs to register: fixed image (on the left) and moving image (on the right). Figure 2. Intensity based monomodal ‘similarity’ registration with gradient descent optimizer. Figure 3. ‘Similarity’ registration with 2D phase correlation algorithm. Figure 4. ‘Similarity’ registration using the automatic detection of SURF (Speeded-Up Robust Features). Figure 5. ‘Similarity’ registration based on the algorithm of automatic detection and matching MSER features (Maximally Stable Extremal Regions). Figure 6. ‘Similarity’ registration with the Fiji (ImageJ) plugin Linear Stack Alignment with SIFT. Figure 7. ‘Similarity’ registration with the Fiji (ImageJ) plugin Register Virtual Stack Slices aided by automatically extracted SIFT features (Scale Invariant Feature Transform). Figure 8. Examples of ‘similarity’ registration with the Fiji (ImageJ) plugin Descriptor-based registration (2d/3d) using SPIM (Selective Plane Illumination Microscope) method. [file 13007_2019_401_MOESM1_ESM.pdf]

Additional file 1

Illustration of the results of tests for automatic registration of fluorescence images.

During the tests carried out with state of art registration methods the fixed image was acquired as the first in the C_3 plant stack (just after pathogen inoculation) of Y(NO) fluorescence images, and one exemplary image of moving type (Fig. 1) and the results of registration test in Figs. 2-5 respectively.

The popular automatic registration methods compare intensity patterns with some correlation metrics (intensity-based) [1, 2] or search in the fixed and moving images for the correspondence between selected image features such as points, lines, and contours (feature-based) [3]. Most of the 'similarity' mapping registration methods can be verified by the *Registration Estimator* application available in Matlab 2017b environment including *Image Processing Toolbox* and *Computer Vision System Toolbox*.

The images obtained in Walz fluorometer were 8-bit intensity images coloured with the predefined, built-in palette, so the monomodal intensity registration [2, 4] was applied to them (Fig. 2). This registration process typically diverges with default optimization parameters and the convergence can be achieved at the cost of additional image smoothing or reducing maximum step of the optimization process. This result is stopping the registration far from the proper alignment both in orientation and scale. The new strong intensity characteristics appearing because of the progressive infection makes the image very difficult to register. Similar effects were obtained with the coarse intensity-based registration by phase correlation method [5] (Fig. 3). The reason for such behaviour of automatic image registration is the lack of regions with strongly distinctive features to be matched in moving and fixed image. Consequently also feature-based 'similarity' registrations with automatically applying SURF (*Speeded-Up Robust Features*) [6], FAST (*Features from Accelerated Segment Test*) [7], BRISK (*Binary Robust Invariant Scalable Keypoints*) [8], MSERF (*Maximally Stable Extremal Regions*) [9], Harris [10], or MinEigen (*Minimum Eigenvalue*) [11] features prevent correct registration, when applying default feature matching of high quality in fixed and moving images (Fig. 4, 5). Significant lowering of the required quality allows for coarse alignment of some image pairs, but at the cost of its poor quality.

The review of other registration applications can be found in the Fiji (ImageJ) environment [12]. The tested methods from this package came from the plugins: *Linear Stack Alignment with* SIFT (*Scale Invariant Feature Transform*), *Register Virtual Stack Slices* [13], and *Descriptor-based registration* (*2d/3d*) based on the SIPM (*Selective Plane Illumination Microscope*) registration algorithm [14, 15]. Examples of performing these methods on PAM fluorescence images are shown in Figs. 6-8. We found that fluorescence images cannot be properly registered with this method. Neither high-quality SIFT features nor SPIM features in *Descriptor-based registration* can be extracted from these images especially those that match each other in pairs of images. The adjustment of many available plugin parameters does not improve registration results because of dynamic changes in the content of the images caused by infection of the leaf tissue. Also, the minimization of intensity error seems to be ill conditioned and does not provide a good registration.

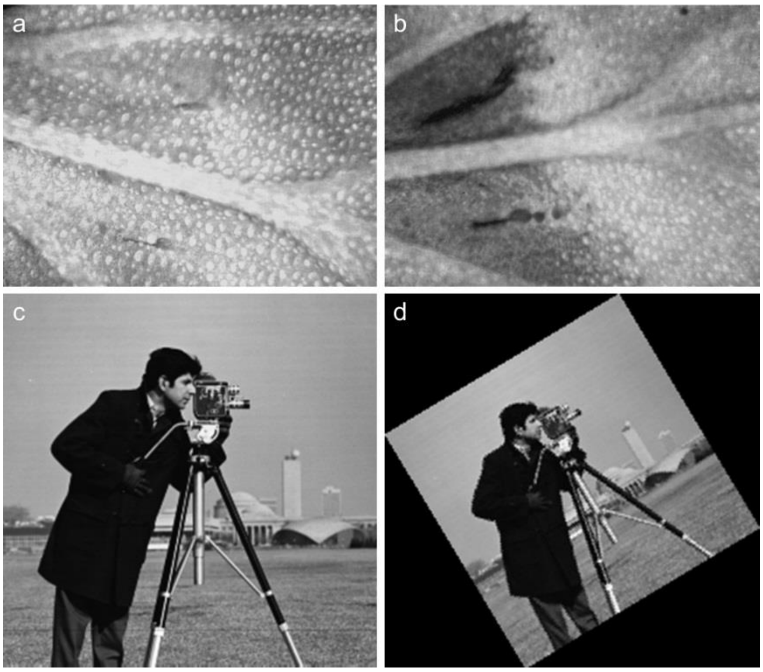

Figure 1 Input image pairs to register: fixed image (on the left) and moving image (on the right). a) C_3 ice-plant leaf image of Y(NO) fluorescence parameter just after (0 h) inoculation with *Botrytis cinerea*, b) Y(NO) image of the same leaf 9 hours after inoculation, c) reference 'cameraman' image, d) 'cameraman' image after rotation and scaling (before registration).

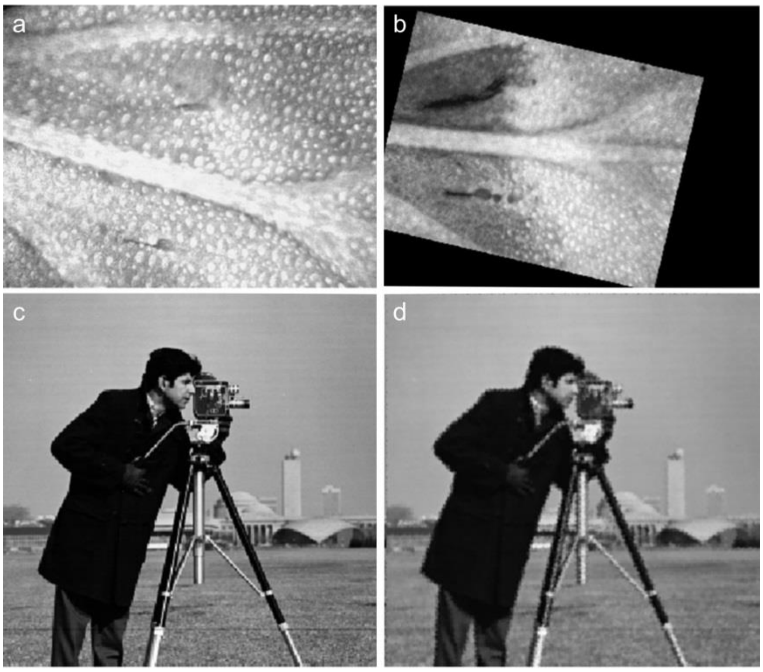

Figure 2 Intensity based monomodal 'similarity' registration with gradient descent optimizer. a) and c) are fixed images of C_3 ice-plant leaf and 'cameraman' respectively, as in Fig. 1, b) poorly registered Y(NO) image from Fig. 1b, d) registered 'cameraman' image from Fig. 1d. The 'cameraman' image basic visible features (despite their deformation) like intensity co-occurrences, corners, edges is registered properly.

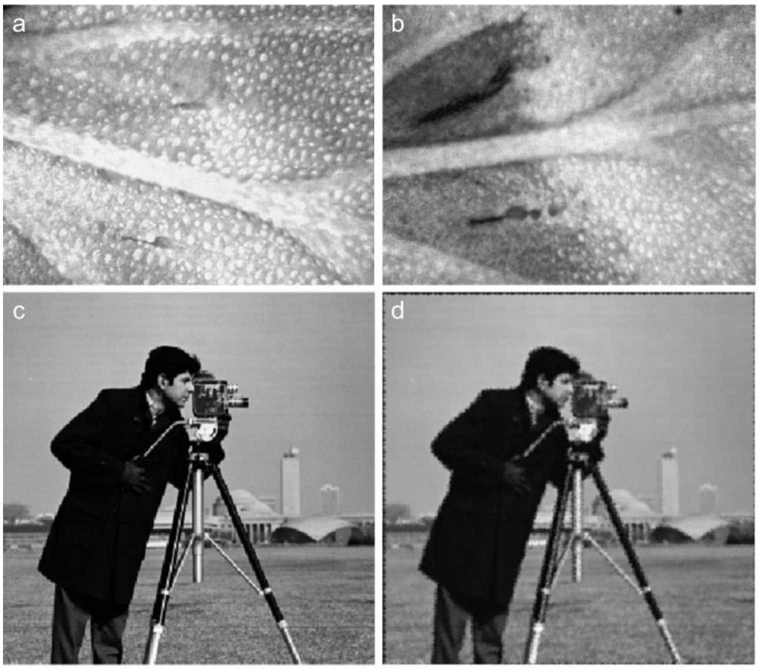

Figure 3 'Similarity' registration with 2D phase correlation algorithm. a) and c) are fixed images of C_3 ice-plant leaf and 'cameraman' respectively, as in Fig. 1, b) incorrectly registered Y(NO) image from Fig. 1b, d) registered 'cameraman' image from Fig. 1d.

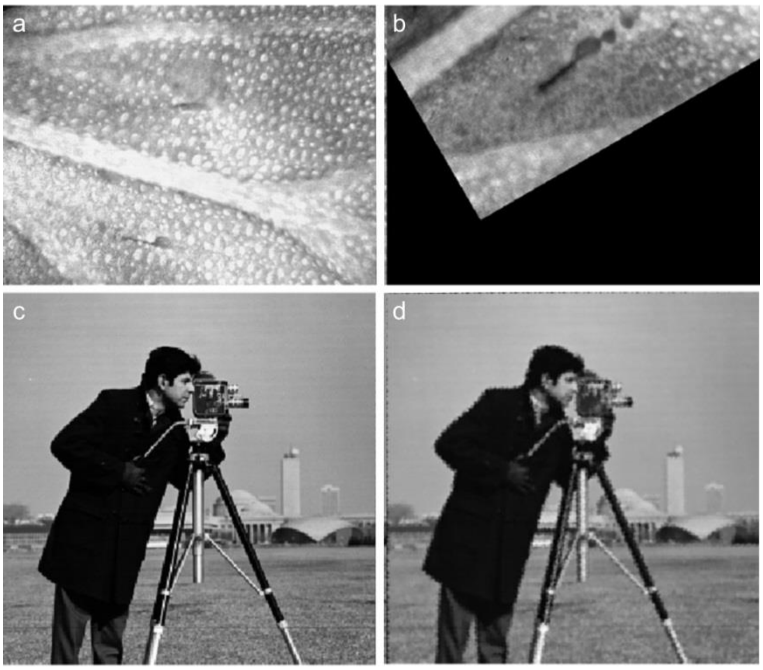

Figure 4 'Similarity' registration using the automatic detection of SURF (*Speeded-Up Robust Features*). a) C_3 fluorescence ice-plant leaf image of Y(NO) feature just after inoculation (fixed image), b) wrongly registered Y(NO) image from Fig. 1b, c) fixed registration image 'cameraman', d) moving 'cameraman' from Fig. 1d after registration.

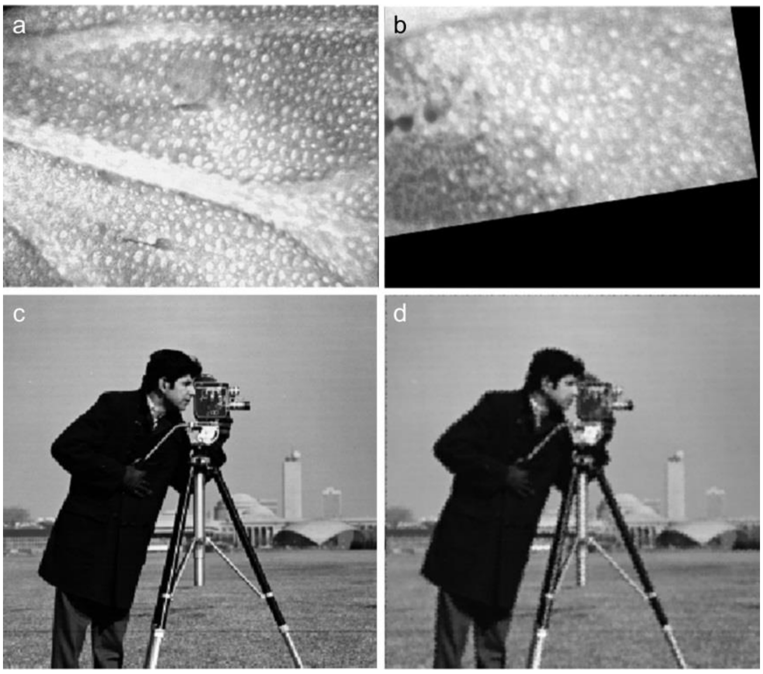

Figure 5 'Similarity' registration based on the algorithm of automatic detection and matching MSER features (*Maximally Stable Extremal Regions*). a) C_3 fluorescence ice-plant leaf image of Y(NO) feature just after inoculation (fixed image), b) incorrectly registered Y(NO) image from Fig. 1b, c) fixed registration image 'cameraman', d) moving 'cameraman' from Fig. 1d after registration.

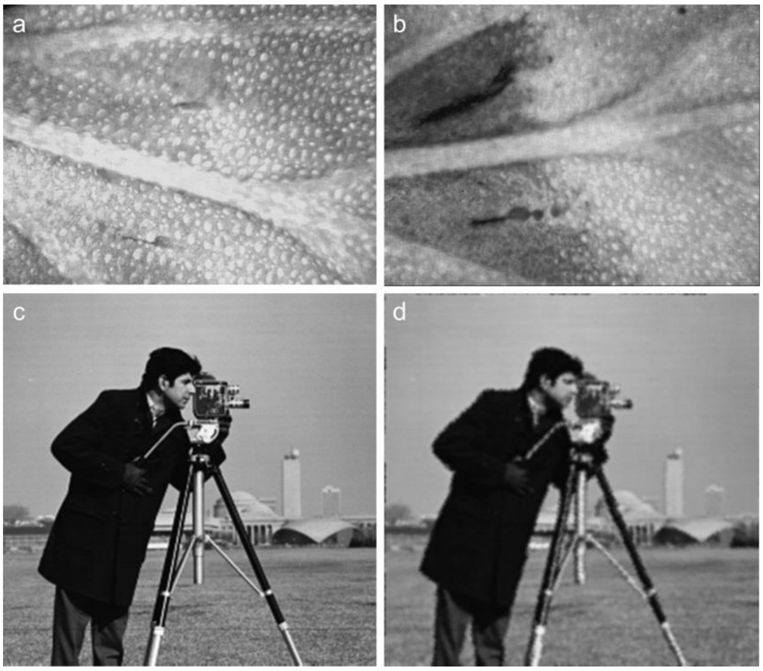

Figure 6 'Similarity' registration with the Fiji (ImageJ) plugin *Linear Stack Alignment with SIFT*. This plugin applying the algorithm of detection and matching SIFT features (*Scale Invariant Feature Transform*). a) C_3 fluorescence ice-plant leaf image of Y(NO) feature just after inoculation (fixed image), b) poorly registered Y(NO) image from Fig. 1b, c) fixed registration image 'cameraman', d) moving 'cameraman' from Fig 1d after registration.

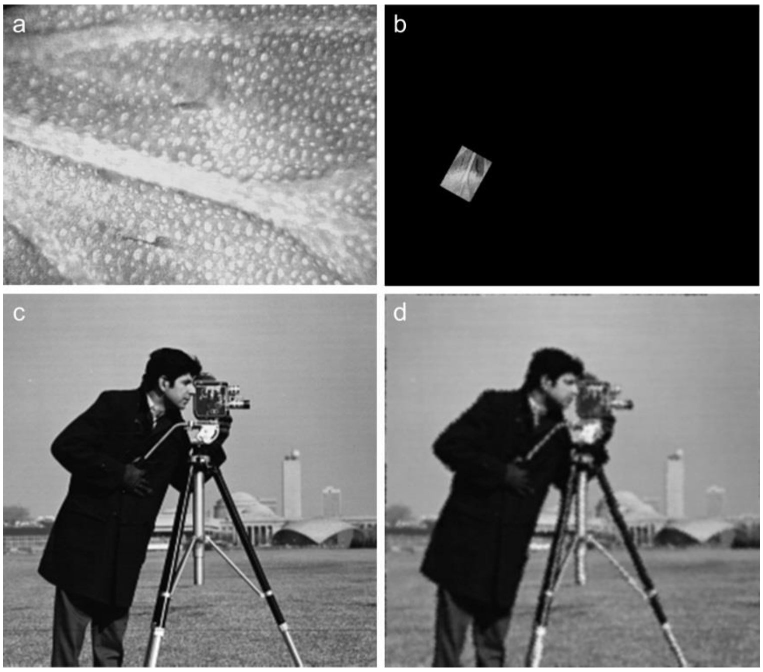

Figure 7 ‘Similarity’ registration with the Fiji (ImageJ) plugin *Register Virtual Stack Slices* aided by automatically extracted SIFT features (*Scale Invariant Feature Transform*). a) C_3 fluorescence ice-plant leaf image of Y(NO) feature just after inoculation (fixed image), b) incorrectly registered Y(NO) image from Fig. 1b, c) fixed registration image ‘cameraman’, d) moving ‘cameraman’ from Fig. 1d after registration.

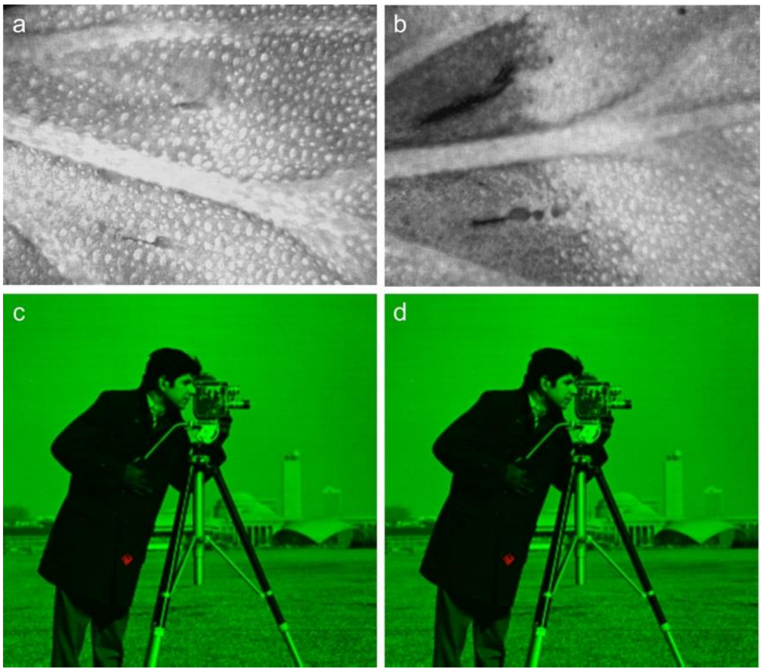

Figure 8 Examples of ‘similarity’ registration with the Fiji (ImageJ) plugin *Descriptor-based registration* (*2d/3d*) using SPIM (*Selective Plane Illumination Microscope*) method. a) C_3 fluorescence ice-plant leaf image of Y(NO) feature just after inoculation (fixed image), b) incorrectly registered Y(NO) image from Fig. 1b, c) fixed registration image ‘cameraman’, d) moving ‘cameraman’ from Fig. 1d after registration.

References to Additional file 1

1. Goshtasby A. 2-D and 3-D image registration for medical, remote sensing, and industrial applications. Wiley Press; 2005.
2. The MathWorks. Intensity-Based Automatic Image Registration. https://www.mathworks.com/help/images/intensity-based-automatic-image-registration.html. Accessed 10 Sept 2018.
3. Papademetris X, Jackowski AP, Schultz RT, Staib LH, Duncan JS. Integrated intensity and point-feature nonrigid registration. Lect Notes in Comput Sci. 2004; doi:10.1007/978-3-540-30135-6_93.
4. The MathWorks. imregconfig. Configurations for intensity-based registration. https://www.mathworks.com/help/images/ref/imregconfig.html. Accessed 10 Sept 2018.
5. Reddy BS, Chatterji BN. An FFT-based technique for translation, rotation, and scale-invariant image registration, IEEE Trans Imag Proc. 1996; 5(8):1266-1271.
6. Bay H, Ess A, Tuytelaars T, Van Gool L. SURF: speeded up robust features. Comput Vis Image Underst. 2008; 110(3):346–359.
7. Rosten E, Drummond T. Fusing points and lines for high performance tracking. Proc of the IEEE International Conference on Computer Vision. 2005; 2:1508–1511.
8. Leutenegger S, Chli M, Siegwart R. BRISK: binary robust invariant scalable keypoints. Proc of the IEEE International Conference, ICCV. 2011.
9. Nister D, Stewenius H. Linear time maximally stable extremal regions. Lect Notes in Comput Sci, 10th European Conference on Computer Vision, Marseille, France. 2008; 5303:183–196.
10. Harris C, Stephens M. A combined corner and edge detector. Proc of the 4th Alvey Vision Conf. 1988:147-151.
11. Shi J, Tomasi C. Good features to track. Proc of the IEEE Conference on Computer Vision and Pattern Recognition. 1994:593–600.
12. Schindelin J, Arganda-Carreras I, Frise E. et al. Fiji: an open-source platform for biological-image analysis. Nat Meth. 2012; doi:10.1038/nmeth.2019.
13. Lowe DG. Distinctive image features from scale-invariant keypoints. Int J Comput Vis. 2004; 60(2):91–110. doi:10.1023/B:VISI.0000029664.99615.94.
14. Preibisch S, Saalfeld S, Schindelin J, Tomancak P. Software for bead-based registration of selective plane illumination microscopy data. Nat Meth. 2010; 7(6):418–419.
15. Preibisch S, Amat F, Stamataki E, Sarov M, Singer RH, Myers E, Tomancak P. Efficient Bayesian-based multiview deconvolution. Nat Meth. 2014; 11(6):645–648.
