# Supplementary material for: Spatial referencing of chlorophyll fluorescence images for quantitative assessment of infection propagation in leaves demonstrated on the ice plant: Botrytis cinerea pathosystem
Source: Plant Methods. 2019 Feb 20;15:18. doi: 10.1186/s13007-019-0401-4 (PMC6381734; doi:10.1186/s13007-019-0401-4)
Supplement: Supplementary file 2 — Additional file 2. The values of fluorescence parameters Y(NO), Fv/Fm and NPQ obtained from ice-plant leaf images without registration. Figure 1. The fluorescence parameter Y(NO) value changes in C3 and CAM common ice plant leaves obtained from ice-plant leaf images without registration. Figure 2. The fluorescence parameter Fv/Fm value changes in C3 and CAM common ice plant leaves obtained from ice-plant leaf images without registration. Figure 3. The fluorescence parameter NPQ value changes in C3 and CAM common ice plant leaves obtained from ice-plant leaf images without registration. [file 13007_2019_401_MOESM2_ESM.pdf]

Additional file 2

The values of fluorescence parameters Y(NO), F_v/F_m and NPQ obtained from ice-plant leaf images without registration.

The fluorescence parameter values are computed at three locations and along the line distance L_1 and L_2 depicted in Fig. 9 of the main manuscript. The measurement points and lines are located properly only on reference image (0 h after *Botrytis cinerea* inoculation) at: 1) mesophyll at the site of inoculation, 2) mesophyll without injury, and 3) midrib, L_1 - mesophyll at the site of inoculation, L_2 - midribs. The values for ice-plant leaf images taken 3-72 h after inoculation are retrieved from incompatible parts of the leaf, which makes the plots unsuitable for interpreting spatiotemporal changes over a sample area.

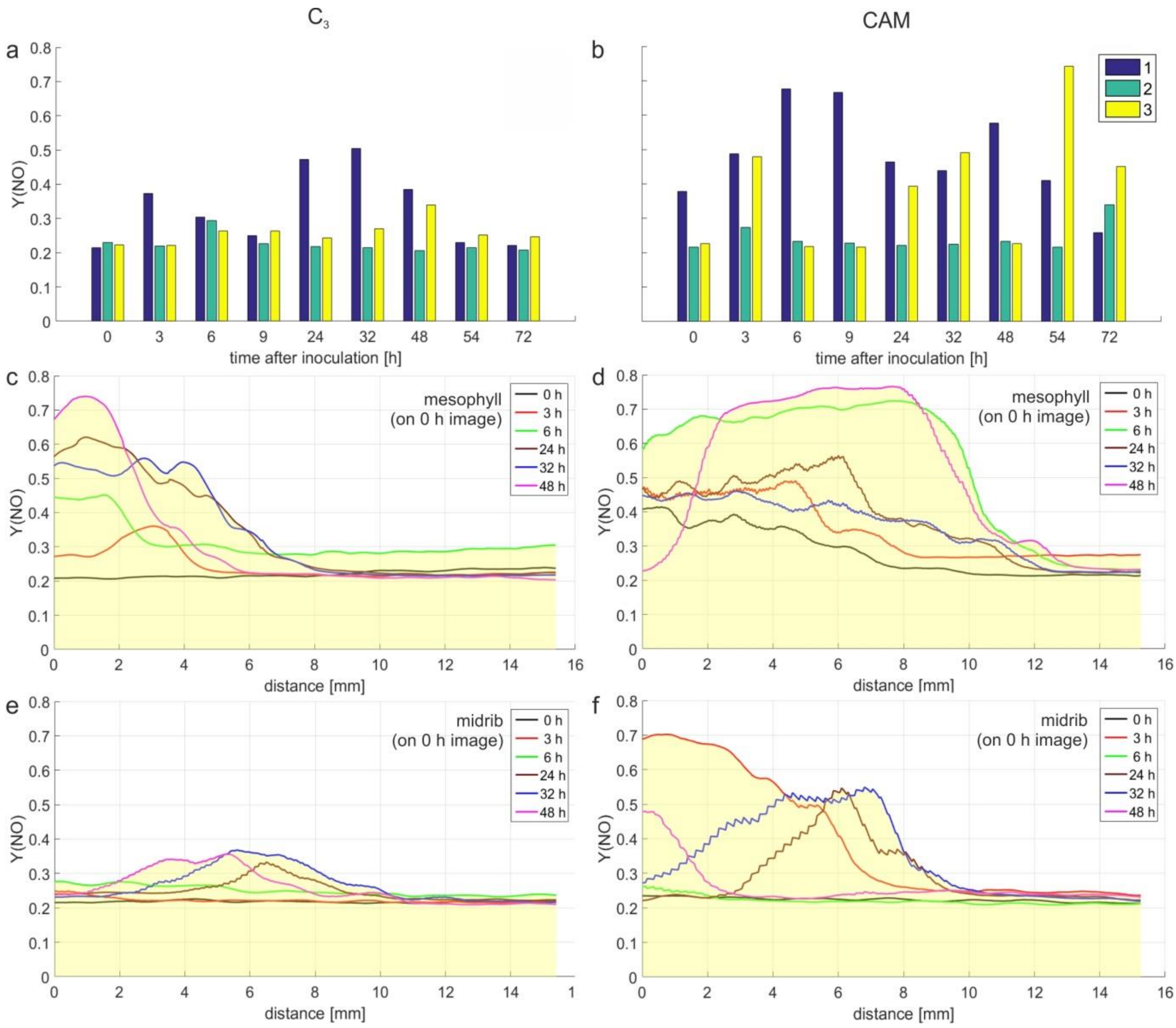

Figure 1 The fluorescence parameter Y(NO) value changes in C_3 and CAM common ice plant leaves obtained from ice-plant leaf images without registration.

Y(NO) values are computed at three locations: 1) mesophyll at the site of inoculation, 2) mesophyll without injury and 3) midrib (**a**-**b**), and along the line distance L_1 - mesophyll at the site of inoculation (**c**-**d**) and L_2 - midribs (**e**-**f**).

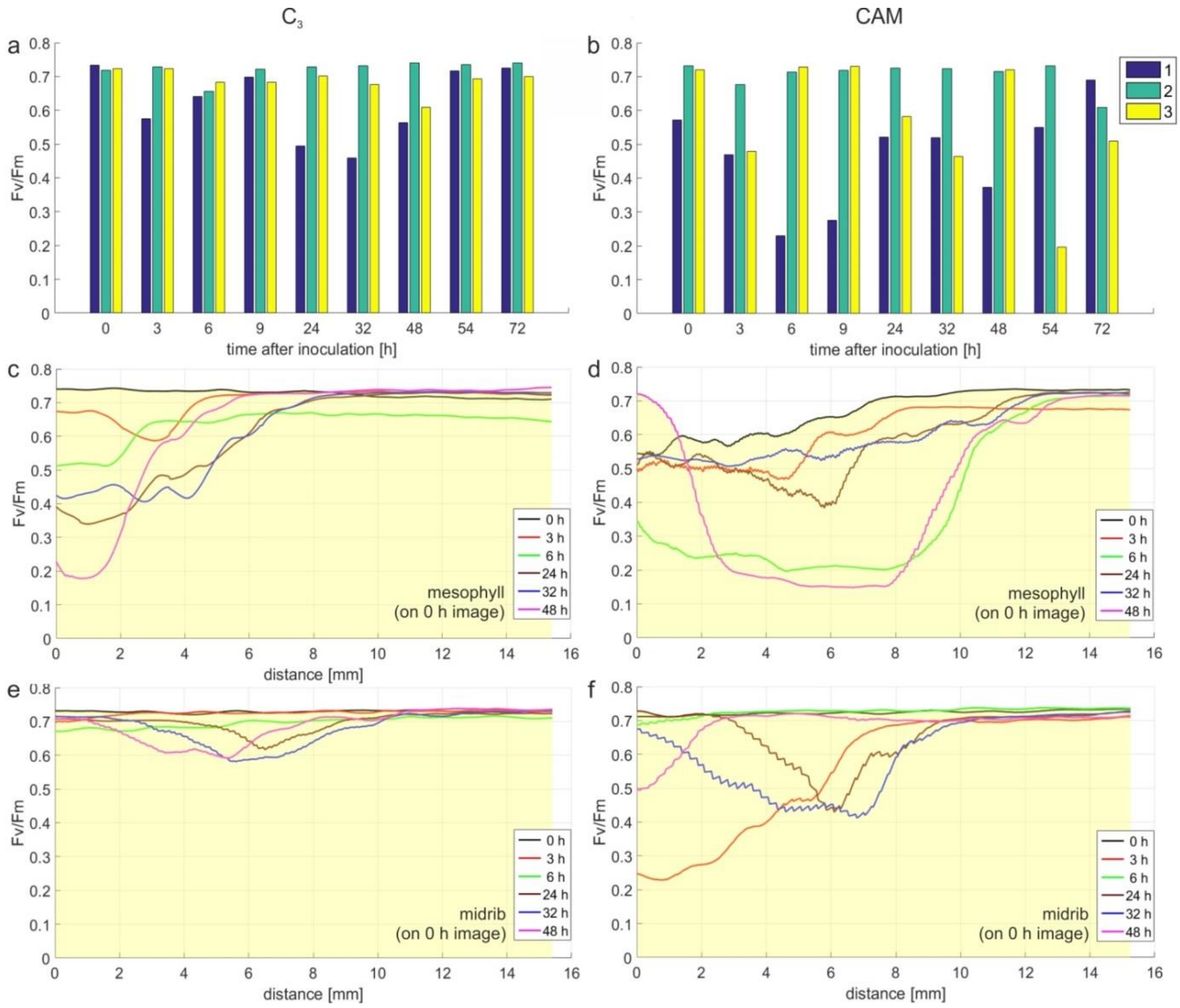

Figure 2 The fluorescence parameter F_v/F_m value changes in C_3 and CAM common ice plant leaves obtained from ice-plant leaf images without registration.

F_v/F_m values are computed at three locations: 1) mesophyll at the site of inoculation, 2) mesophyll without injury and 3) midrib (**a**-**b**), and along the line distance L_1 - mesophyll at the site of inoculation (**c**-**d**) and L_2 - midribs (**e**-**f**).

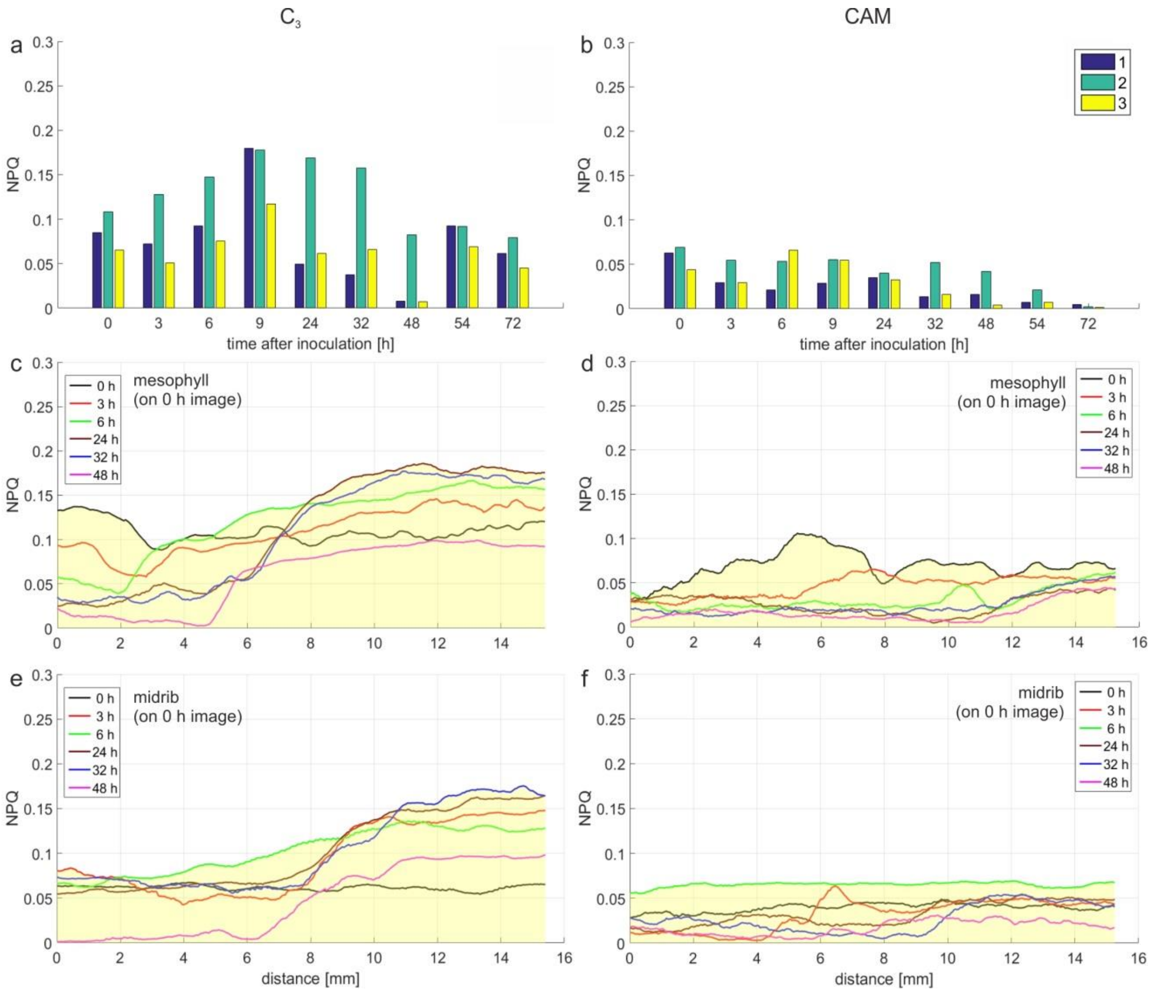

Figure 3 The fluorescence parameter NPQ value changes in C_3 and CAM common ice plant leaves obtained from ice-plant leaf images without registration.

NPQ values are computed at three locations: 1) mesophyll at the site of inoculation, 2) mesophyll without injury and 3) midrib (**a**-**b**), and along the line distance L_1 - mesophyll at the site of inoculation (**c**-**d**) and L_2 - midribs (**e**-**f**).
